# New Furanocembranoids from *Briareum violaceum*

**DOI:** 10.3390/md17040214

**Published:** 2019-04-05

**Authors:** Pin-Chang Huang, Wen-Sou Lin, Bo-Rong Peng, Yu-Chia Chang, Lee-Shing Fang, Guo-Qiang Li, Tsong-Long Hwang, Zhi-Hong Wen, Ping-Jyun Sung

**Affiliations:** 1 Graduate Institute of Marine Biology, National Dong Hwa University, Pingtung 94450, Taiwan; foter25632@gmail.com; 2 Department of Planning and Research, National Museum of Marine Biology and Aquarium, Pingtung 94450, Taiwan; pengpojung@gmail.com; 3 Department of Neurology, Kaohsiung Armed Forces General Hospital, Kaohsiung 80284, Taiwan; linvincent1009@gmail.com; 4 Department of Marine Biotechnology and Resources, National Sun Yat-sen University, Kaohsiung 80424, Taiwan; 5 Research Center for Chinese Herbal Medicine, Research Center for Food and Cosmetic Safety, Graduate Institute of Healthy Industry Technology, College of Human Ecology, Chang Gung University of Science and Technology, Taoyuan 33303, Taiwan; jay0404@gmail.com (Y.-C.C.); htl@mail.cgu.edu.tw (T.-L.H.); 6 Center for Environmental Toxin and Emerging-Contaminant Research, Cheng Shiu University, Kaohsiung 83347, Taiwan; lsfang@csu.edu.tw; 7 Super Micro Mass Research and Technology Center, Cheng Shiu University, Kaohsiung 83347, Taiwan; 8 Key Laboratory of Marine Drugs, Chinese Ministry of Education, School of Medicine and Pharmacy, Ocean University of China, Qingdao 266033, China; liguoqiang@ouc.edu.cn.; 9 Laboratory of Marine Drugs and Biological Products, National Laboratory for Marine Science and Technology, Qingdao 266235, China; 10 Graduate Institute of Natural Products, College of Medicine, Chang Gung University, Taoyuan 33302, Taiwan; 11 Chinese Herbal Medicine Research Team, Healthy Aging Research Center, Chang Gung University, Taoyuan 33302, Taiwan; 12 Department of Anaesthesiology, Chang Gung Memorial Hospital, Taoyuan 33305, Taiwan; 13 Graduate Institute of Natural Products, Kaohsiung Medical University, Kaohsiung 80708, Taiwan; 14 Research Center for Natural Products and Drug Development, Kaohsiung Medical University, Kaohsiung 80708, Taiwan; 15 Chinese Medicine Research and Development Center, China Medical University Hospital, Taichung 40447, Taiwan

**Keywords:** *Briareum violaceum*, briaviodiol, briaviotriol, anti-inflammatory, iNOS

## Abstract

Three new furanocembranoids—briaviodiol F (**1**) and briaviotriols A (**2**) and B (**3**)—along with a known analogue, briaviodiol A (**4**), were obtained from a cultured-type octocoral *Briareum violaceum*. The structures of cembranoids **1**–**3** were elucidated by using spectroscopic methods. In vitro study demonstrated that compounds **2** and **4** exerted inhibition effects on inducible nitric oxide synthase (iNOS) release from RAW 264.7, a macrophage cell line that originated from a mouse monocyte macrophage, stimulated with lipopolysaccharides.

## 1. Introduction

**Figure 1:**
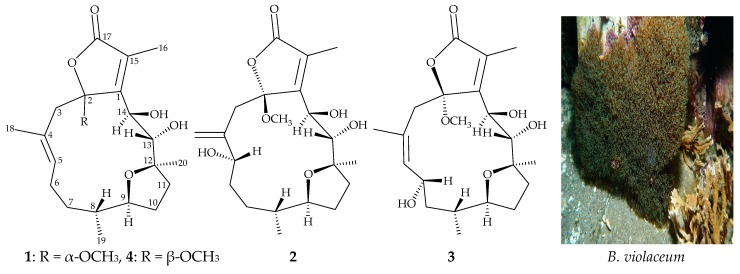
Structures of briaviodiol F (**1**), briaviotriols A (**2**) and B (**3**), and briaviodiol A (**4**), and a picture of the octocoral *B. violaceum*.

*Briareum violaceum* (Quoy and Gaimard, 1883) is a soft coral of the family Briareidae [1,2], which has been found to contain cembrane-type diterpenoids in abundance [3,4,5,6,7,8,9,10]. Diterpenoids of this type have been reported to have complicated structures and possess a variety of bioactivities [3,4,5,6,7,8,9,10]. Recently, in our research into the chemical constituents and properties of a cultured octocoral *B. violaceum*, we have isolated three previously unreported furanocembranoids— briaviodiol F (**1**), and briaviotriols A (**2**) and B (**3**)—along with a known analogue, briaviodiol A (**4**) [9] (Figure 1). A pro-inflammatory suppression assay was employed to assess the activities of these isolated compounds against the release of inducible nitric oxide synthase (iNOS) from macrophage cells.

## 2. Results and Discussion

Briaviodiol F (**1**) was isolated as a colorless oil. Compound **1** displayed a pseudomolecular ion at *m*/*z* 403.20886 in the (+)-HRESIMS, which indicated its molecular formula was C_21_H_32_O_6_ (calcd. for C_21_H_32_O_6_ + Na, 403.20911), suggesting six degrees of unsaturation. Additionally, IR absorptions at 3497 and 1754 cm^–1^ indicated that **1** contained hydroxy and ester groups. As shown in Table 1, DEPT and ^13^C NMR spectra indicated that a suite of ^13^C resonances at δ_C_ 172.1 (C-17), 154.3 (C-1), 127.2 (C-15), 109.5 (C-2), and 9.2 (CH_3_-16) were due to an α-methyl-γ-butenolide moiety by comparison with the data of known cembranoids briaviodiol A (**4**) [9] and pachyclavulariolide F [6]. Moreover, resonances at δ_C_ 127.1 (C-4) and 135.1 (CH-5), and the olefinic proton at δ_H_ 5.30 (1H, dd, *J* = 8.0, 5.6 Hz, H-5) (Table 1), indicated an additional unsaturated functionality, suggesting the presence of a trisubstituted olefin. In the HSQC spectrum, an sp^2^ carbon (δ_C_ 135.1) correlated with the methine proton (δ_H_ 5.30). This proton had ^3^*J*-correlations with H_2_-6 (δ_H_ 1.93–1.99, 2H, m) in the ^1^H–^1^H COSY spectrum, and had ^3^*J*-correlations with C-3 and C-18 in the HMBC spectrum (Table 1), further confirming the existence of a trisubstituted olefin. In light of the ^1^H and ^13^C NMR data, together with the degrees of unsaturation, **1** was determined as a tricyclic cembrane diterpene.

The ^1^H NMR coupling information in the COSY spectrum of **1** enabled the determination of the proton sequences between H-5/H_2_-6/H_2_-7/H-8/H-9/H_2_-10/H_2_-11 and H-8/H_3_-19 (Table 1). The carbon skeleton of **1** was elucidated based on the key HMBC from H-3β, H-13, H-14, H_3_-16 to C-1; H_2_-3, H-14 to C-2; H_2_-3, H_3_-18 to C-4; H-10β, H-11α, H-13, H-14, H_3_-20 to C-12; H-14, H_3_-16 to C-15; and H_3_-16 to C-17. The presence of a vinyl methyl group on C-4 was supported by HMBC from H_3_-18 to C-3, C-4, C-5; H-3α (δ_H_ 2.78) to C-18 and H-5 to C-18. Furthermore, HMBC from OH-13 to C-13, C-14 and OH-14 to C-1, C-13, C-14 suggested the existence of hydroxy groups at C-13 and C-14, respectively. Therefore, the methoxy group was on C-2, since the HMBC spectrum exhibited a correlation between the singlet at δ_H_ 3.39 (OMe) and C-2 (δ_C_ 109.5). Taking into account the molecular formula, the remaining oxygen atom must be part of the tetrahydrofuran ring located between C-9 and C-12.

**Figure 2:**
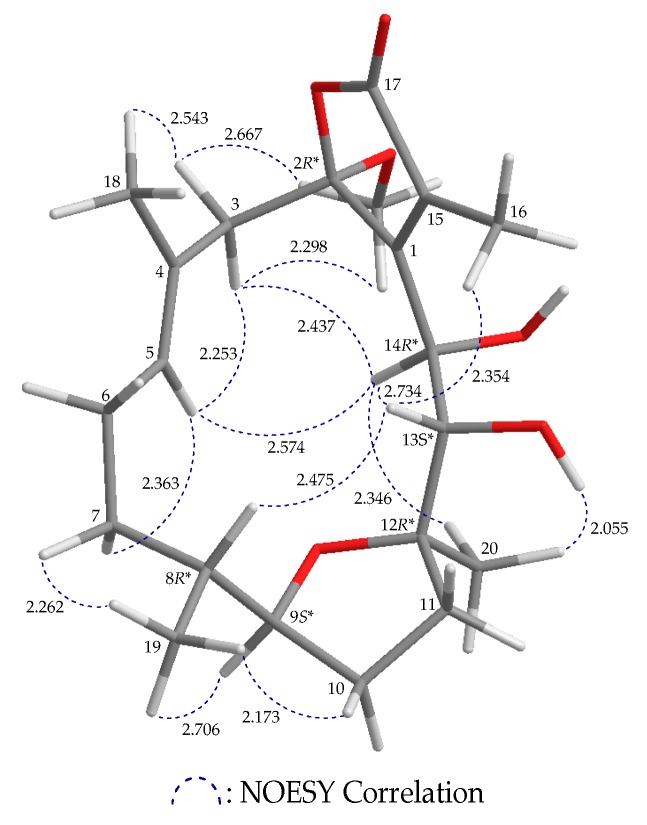
Computer-depicted model drawing of **1** and calculated distances (unit = Å) between protons with main NOESY correlations.

Based on NOESY correlations and further information provided by MM2 forcefield calculations [11], the relative stereochemistry of **1** with the stable conformation is shown in Figure 2 (Supplementary Figures S1–10). When H-9 was α-oriented in **1**, a correlation between H-9 and H_3_-19 was observed, suggesting that these protons were on the α-face, and H-8 was β-oriented. H-8 correlated with H-13, and the hydroxy proton OH-13 correlated with H_3_-20, suggesting that the hydroxy group at C-13 and the Me-20 at C-12 were α-oriented. H-14 exhibited a NOESY correlation with H-13, and no coupling constant was detected between H-13 and H-14 in the ^1^H NMR spectrum, implying that the dihedral angle located between H-13 and H-14 was about 90°, and the 14-hydroxy group was β-oriented. Correlations between H-5 and H-3, and H-14 and H-3 (δ_H_ 2.78) suggested that this proton is α, and the proton at δ_H_ 3.04 is 3β. Additionally, the proton signal of a methoxy group displayed NOESY correlations with both H-3α/β, which indicated that the methoxy group at C-2 was α-oriented. H_3_-18 was found to show a NOESY correlation with H-3β, but not with H-5, and H-5 was shown to be correlated with H-3α and H-14, which suggested an *E*-configuration of the C-4/5 double bond. The aforementioned results enabled establishment of the relative configuration of **1**, and therefore its stereogenic carbons were assigned as 2R*,8R*,9S*,12R*,13S*, 14R*.

Briaviotriol A (**2**) was found to have the molecular formula C_21_H_32_O_7_, as established by (+)-HRESIMS at *m*/*z* 419.20377 (calcd. for C_21_H_32_O_7_ + Na, 419.20402). The ^1^H and ^13^C NMR spectra of **2** were very similar to those of **1**. Comparison between the ^1^H and ^13^C NMR data of **2** (Table 2) and those of **1** suggested that the double bond is located between C-4 and C-18 in **2** instead of C-4 and C-15 in **1**. HMBC from H_2_-18 to C-3, C-4, C-5; and from H_2_-3 and H-5 to C-18, corroborated the existence of an exocyclic double bond at C-4. In the HSQC spectrum, an oxymethine carbon (δ_C_ 69.1) correlated with the methine proton (δ_H_ 4.57), and this proton had ^3^*J*-correlations with H_2_-6 (δ_H_ 1.81, 1H, m and 1.93, 1H, m) in the ^1^H–^1^H COSY spectrum, demonstrating that a hydroxy group was attached to C-5.

**Figure 3:**
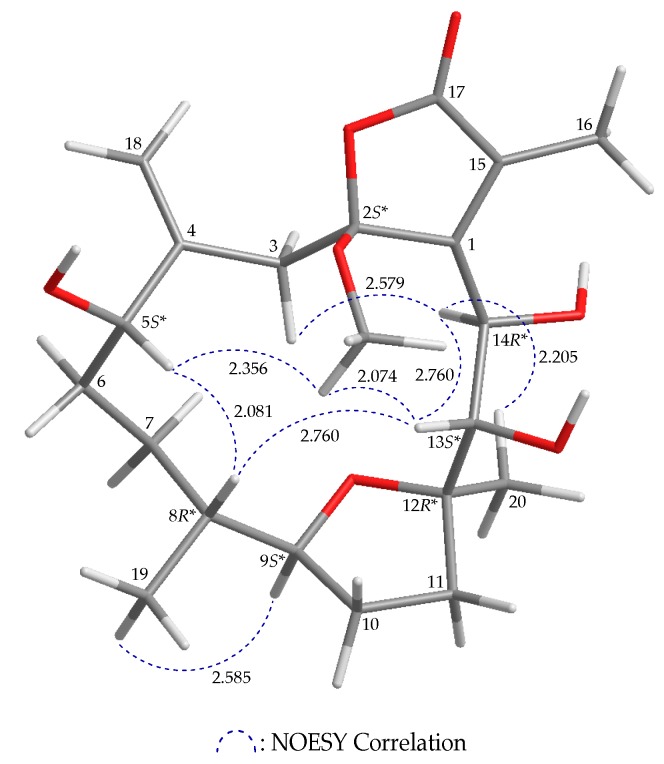
Computer-depicted model drawing of **2** and calculated distances (unit = Å) between protons with main NOESY correlations.

The stereochemistry of **2** was established from the correlations observed in the NOESY spectrum (Figure 3 and Supplementary Figures S2–10). In addition, in the NOESY spectrum H-9 was correlated with H_3_-19, which suggested that these protons were positioned on the same face and were assigned as α protons, as H-8 was β-oriented. H-13 correlated with H-8 and H-14, but no coupling between H-13 and H-14 was observed, demonstrating that the hydroxy groups at C-13 and C-14 were α- and β-oriented, respectively. Correlations between an oxygen-bearing methyl (δ_H_ 3.26) and H-13 suggested that the C-2 methoxy group was situated on the β face. Additionally, correlation between H-5 and H-8 supported a β-orientation of H-5. Based on the aforementioned results, the relative configurations of the stereogenic carbons of **2** were determined as 2S*,5S*,8R*, 9S*,12R*,13S*,14R*.

**Figure 4:**
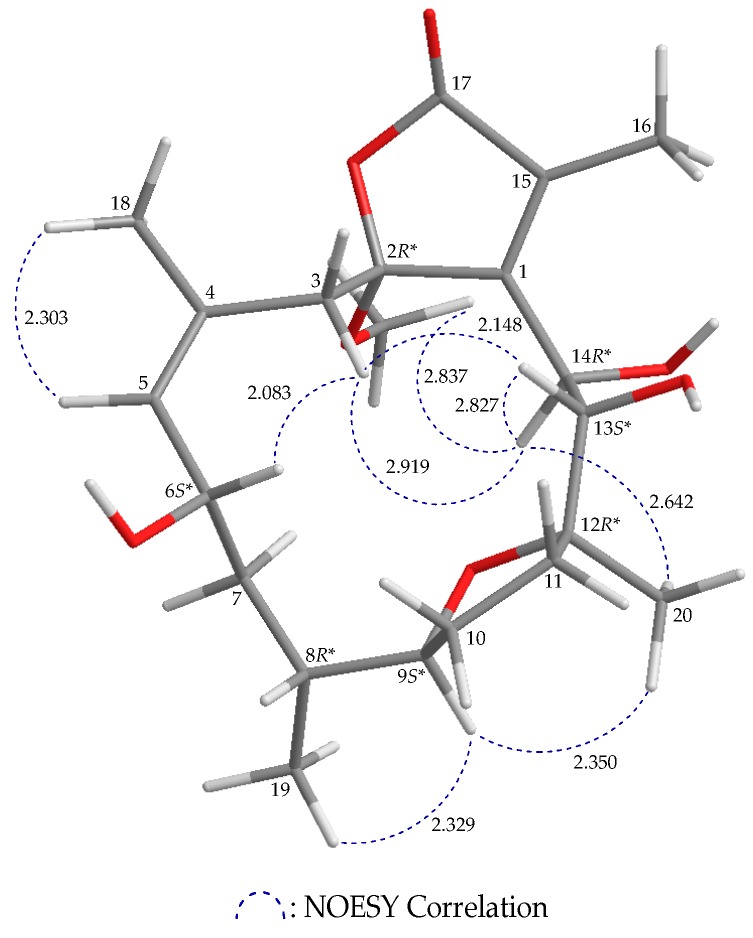
Computer-depicted model drawing of **3** and calculated distances (unit = Å) between protons with main NOESY correlations.

Compound **3** has a molecular formula C_21_H_32_O_7_ according to its (+)-HRESIMS *m*/*z* 419.20399 (calcd. for C_21_H_32_O_8_ + Na, 419.20402). The ^1^H and ^13^C NMR features of **3** resemble those of **1**; comparison of the ^1^H and ^13^C NMR chemical shifts of the sp^2^ methine proton and its respective carbon (δ_H_ 5.29, 1H, d, *J* = 8.4 Hz; δ_C_ 133.5, CH-5), and the sp^2^ quaternary carbon (δ_C_ 131.0, C-4) of **3** (Table 3) with those of **1** (δ_H_ 5.30, 1H, dd, *J* = 8.0, 5.6 Hz; δ_C_ 135.1, CH-5; δ_C_ 127.1, C-4) (Table 1), as well as a NOESY correlation between H-5 and H_3_-18, indicated the *Z*-configuration of the C-4/5 double bond (Figure 4 and Supplementary Figure S3–10). Furthermore, the HSQC spectrum showed that an oxymethine carbon (δ_C_ 68.7) was correlated with a methine proton (δ_H_ 4.86; H-6), and this proton exhibited ^3^*J*-correlations with the olefinic proton H-5 (δ_H_ 5.29) and H_2_-7 (δ_H_ 1.34, 1H, m; 1.80, 1H, m) in the COSY spectrum, which confirmed a hydroxy group at C-6. As H-6 showed a NOESY correlation with H-3β, this suggested that the C-6 hydroxy group was α-oriented. Based on a NOESY experiment (Figure 4 and Supplementary Figures S3–10), **3** was identified to have the stereogenic centers 2R*,6S*,8R*,9S*,12R*,13S*,14R*. Since **3** has never been previously reported, it was named briaviotriol B.

Compound **4** was identified as briaviodiol A (Figure 1), by comparison of its ^1^H and ^13^C NMR data with those in the literature [9].

Using an in vitro pro-inflammatory suppression assay, the effects of **1**–**4** on the release of iNOS protein from lipopolysaccharide (LPS)-stimulated RAW 264.7 macrophage cells were assessed. First, alamar blue cell viability assessment revealed that **1**–**4** did not have significant cytotoxic effects in RAW 264.7 cells. The results of the in vitro pro-inflammatory suppression assay showed that **2** and **4** at 10 μM suppressed the release of iNOS to 67.7 and 61.9%, respectively, when compared with results of the cells stimulated with only LPS (Table 4). Compound **1** showed no suppression effect on iNOS release.

## 3. Experimental Section

### 3.1. General Experimental Procedures

The JEOL NMR spectrometer (model ECZ400S, Tokyo, Japan) was used to record the spectra with the solvent peak of CHCl_3_ (δ_H_ 7.26 ppm) and CDCl_3_ (δ_C_ 77.1 ppm) as internal references for ^1^H NMR and ^13^C NMR, respectively. ESIMS and HRESIMS were obtained from the Bruker mass spectrometer with 7 Tesla magnets (model: SolariX FTMS system) (Bremen, Germany). Column chromatography, IR spectra and optical rotation were performed according to our earlier research [10].

### 3.2. Animal Material

Specimens of *B. violaceum* used for this study were collected in December 2016 from the cultivation tank (capacity = 270 tons) at the National Museum of Marine Biology and Aquarium (NMMBA) in Southern Taiwan. For its identification, this coral species was compared to reliable sources published earlier [1,2]. A voucher specimen was deposited in the NMMBA (voucher no.: NMMBA-CSC-005).

### 3.3. Extraction and Isolation

Sliced bodies (wet/dry weight = 358.7/144.5 g) of the coral specimen were prepared and extracted with a 1:1 mixture of MeOH and CH_2_Cl_2_ to give 17.2 g of crude extract which was partitioned between EtOAc and H_2_O to obtain 6.3 g of the EtOAc extract. The EtOAc extract was then applied onto a silica gel column and eluted with gradients of *n*-hexane/EtOAc (100% *n*-hexane−100% EtOAc, stepwise), to furnish 14 fractions (fractions: A−N). Fraction G was further chromatographed on a silica gel column and eluted with gradients of *n*-hexane/Me_2_CO (20:1−100% Me_2_CO, stepwise) to afford 11 subfractions (fractions: G1−G11). Fraction G4 was applied onto a silica gel column and eluted with gradients of *n*-hexane and Me_2_CO (20:1−100% Me_2_CO, stepwise) to give 12 subfractions (fractions: G4A−G4L). Afterwards, fraction G4E was then separated by normal-phase HPLC (NP-HPLC) using a mixture of *n*-hexane and Me_2_CO (5:1) as solvent to obtain 5 subfractions (fractions: G4E1−G4E5). Then, fraction G4E1 was separated by NP-HPLC using a mixture of CH_2_Cl_2_ and Me_2_CO (with volume: volume = 80:1; at a flow rate = 3.0 mL/min) to afford **1** (62.7 mg). Fraction G4H was separated by NP-HPLC using a mixture of *n*-hexane and Me_2_CO (with volume: volume = 4:1; at a flow rate = 2.0 mL/min) to afford **4** (17.0 mg). Fraction G4J was repurified by NP-HPLC using a mixture of *n*-hexane and Me_2_CO (with volume: volume = 3:1; at a flow rate = 2.0 mL/min) to afford **3** (0.9 mg). Fraction G8 was separated by NP-HPLC using a mixture of *n*-hexane and Me_2_CO (3:1) to obtain 6 subfractions G8A−G8F. Fraction G8F was repurified by reverse-phase HPLC (RP-HPLC) using a mixture of MeCN and H_2_O (with volume: volume = 1:1; at a flow rate = 1.0 mL/min) to yield **2** (1.2 mg).

Briaviodiol F (**1**): Colorless oil; ${\left[\alpha \right]}_{D}^{21}$ +223 (*c* 1.48, CHCl_3_); IR (neat) ν_max_ 3497, 1754 cm^−1^; ^1^H and ^13^C NMR data (see Table 1); ESIMS: *m*/*z* 403 [M + Na]^+^; HRESIMS: *m*/*z* 403.20886 (calcd. for C_21_H_32_O_6_ + Na, 403.20911).

Briaviotriol A (**2**): Colorless oil; ${\left[\alpha \right]}_{D}^{22}$ −68 (*c* 0.06, CHCl_3_); IR (neat) ν_max_ 3424, 1749 cm^−1^; ^1^H and ^13^C NMR data (see Table 2); ESIMS: *m*/*z* 419 [M + Na]^+^; HRESIMS: *m*/*z* 419.20377 (calcd. for C_21_H_32_O_7_ + Na, 419.20402).

Briaviotriol B (**3**): Colorless oil; ${\left[\alpha \right]}_{D}^{23}$ −39 (*c* 0.04, CHCl_3_); IR (neat) ν_max_ 3424, 1749 cm^−1^; ^1^H and ^13^C NMR data (see Table 3); ESIMS: *m*/*z* 419 [M + Na]^+^; HRESIMS: *m*/*z* 419.20399 (calcd. for C_21_H_32_O_7_ + Na, 419.20402).

Briaviodiol A (**4**): Colorless crystal; ${\left[\alpha \right]}_{D}^{21}$ −52 (*c* 0.85, CHCl_3_) (Reference [9] ${\left[\alpha \right]}_{D}^{23}$ −31 (*c* 0.14, CHCl_3_)); IR (neat) ν_max_ 3467, 1747 cm^−1^; ^1^H and ^13^C NMR data were found to be in absolute agreement with previous study [9]; ESIMS: *m*/*z* 403 [M + Na]^+^.

### 3.4. Molecular Mechanics Calculations

The molecular models were generated by implementing the MM2 force field [11] in ChemBio 3D Ultra software (ver. 12.0) which was created by CambridgeSoft (PerkinElmer, Cambridge, MA, USA).

### 3.5. In Vitro Anti-Inflammatory Assay

The pro-inflammatory suppression assay was performed using a murine macrophage cell line, RAW 264.7, which was purchased from the American Type Culture Collection (ATCC cell line no. TIB-71; Manassas, VA, USA). Untreated or LPS-induced RAW 264.7 cells were used to determine the anti-inflammatory activities of cembranoids **1**–**4** by assessing the inhibition of pro-inflammatory iNOS release from macrophage cells. The iNOS protein levels were measured by using western blotting analysis [12,13,14]. Briefly, in the control group, macrophages were incubated in compound-free medium with LPS (10 μM) alone for 16 h; and in the cembranoid-treated groups, the cells were pre-treated with cembranoids **1**–**4** (10 μM) for 10 min followed by an LPS challenge for 16 h. After the incubation, cell lysates were collected, and equal amounts of the total protein samples were subjected to western blot analysis. The immunoreactivities were caculated based on the optical densities of the corresponding iNOS bands of each group on the membrane, and the cells with LPS treatment alone were set to be 100%. Viability of macrophage cells of different groups was determined after treatment with alamar blue (Invitrogen, Carlsbad, CA, USA), a chemical of tetrazolium dye that is reduced by living cells to a fluorescent substance. The assay has been shown to have accurate measurement in determining the survival of RAW 264.7 cells [15,16], which is based on a mechanism similar to that of an assay using 3-(4,5-dimethyldiazol-2-yl)-2,5- diphenyltetrazolium bromide. Data analyses were firstly performed using one-way analysis of variance (ANOVA), and further analyzed by the Student-Newman-Keuls post hoc test for multiple comparison. All the data with a *p*-value of < 0.05 were considered as a significant difference. 

## 4. Conclusions

*B. violaceum* has been demonstrated to have a wide structural diversity of interesting diterpenoids that possess various pharmacological properties [17]. This specimen was encrusted on different species of scleractinian hard corals in the Indo-Pacific coral reef system [18]. In our continued study of *B. violaceum*, three previously unreported furanocembranoids **1**–**3** were isolated, together with the previously described briaviodiol A (**4**). In the present study, the anti- inflammatory activities of **1**–**4** were assessed using inhibition of pro-inflammatory iNOS release from macrophages. The results indicated that briaviotriol A (**2**) and briaviodiol A (**4**) showed the most potent suppressive effects on iNOS release.

## Figures and Tables

**Table 1:** ^1^H (400 MHz, CDCl_3_) and ^13^C (100 MHz, CDCl_3_) NMR, COSY, HMBC data for **1**.

Position	δ_H_ (*J* in Hz)	δ_C_, type	COSY	HMBC
1		154.3, C		
2		109.5, C		
3α/β	2.78 d (14.0); 3.04 d (14.0)	44.8, CH_2_		C-1, C-2, C-4, C-5, C-18
4		127.1, C		
5	5.30 dd (8.0, 5.6)	135.1, CH	H_2_-6	C-3, C-18
6	1.93–1.99 m	26.3, CH_2_	H-5, H_2_-7	C-4, C-5, C-7, C-8
7α/β	1.80 m; 1.26 m	34.9, CH_2_	H_2_-6, H-8	C-5, C-6, C-8, C-9, C-19
8	0.68 m	41.4, CH	H_2_-7, H-9, H_3_-19	C-9
9	3.68 ddd (9.6, 9.6, 6.0)	85.2, CH	H-8, H_2_-10	C-7
10α/β	1.51 m; 1.98 m	30.3, CH_2_	H-9, H_2_-11	C-11, C-12
11α/β	1.56 m; 2.38 dd (12.0, 6.4)	36.9, CH_2_	H_2_-10	C-9, C-10, C-12, C-13, C-20
12		84.1, C		
13	3.60 d (10.0)	70.7, CH	OH-13	C-1, C-11, C-12, C-20
14	5.06 s	63.9, CH	-	C-1, C-2, C-12, C-15
15		127.2, C		
16	2.11 s	9.2, CH_3_		C-1, C-15, C-17
17		172.1, C		
18	1.44 s	15.3, CH_3_		C-3, C-4, C-5
19	0.78 d (6.4)	17.3, CH_3_	H-8	C-7, C-8, C-9
20	1.30 s	20.9, CH_3_		C-11, C-12, C-13
OMe-2	3.39 s	51.0, CH_3_		C-2
OH-13	3.14 d (10.0)		H-13	C-13, C-14
OH-14	3.34 s		-	C-1, C-13, C-14

**Table 2:** ^1^H (400 MHz, CDCl_3_) and ^13^C (100 MHz, CDCl_3_) NMR, COSY, HMBC data for **2**.

Position	δ_H_ (*J* in Hz)	δ_C_, type	COSY	HMBC
1		157.9, C		
2		107.8, C		
3α/β	2.67 d (14.4); 3.15 d (14.4)	42.2, CH_2_		C-1, C-2, C-4, C-5, C-18
4		146.4, C		
5	4.57 dd (7.2, 6.0)	69.1, CH	H_2_-6	C-4, C-6, C-18
6/6′	1.81m; 1.93 m	32.2, CH_2_	H-5, H_2_-7	-
7/7′	1.33 m; 1.89 ddd (14.4, 4.8, 4.4)	30.6, CH_2_	H_2_-6, H-8	C-5
8	1.53 m	37.4, CH	H_2_-7, H-9, H_3_-19	-
9	3.65 ddd (8.4, 8.4, 6.0)	85.4, CH	H-8, H_2_-10	-
10/10′	1.52 m; 2.10 m	31.5, CH_2_	H-9, H_2_-11	-
11/11′	1.65 m; 2.16 m	37.2, CH_2_	H_2_-10	C-9, C-10, C-12, C-13, C-20
12		84.4, C		
13	3.50 d (5.6)	75.2, CH	OH-13	-
14	5.18 br s	67.7, CH	OH-14	-
15		130.0, C		
16	2.11 s	10.1, CH_3_		C-1, C-15, C-17
17		171.1, C		
18a/b	5.13 s; 5.30 s	115.6, CH_2_		C-3, C-4, C-5
19	0.85 d (6.4)	16.7, CH_3_	H-8	C-7, C-8, C-9
20	1.29 s	21.8, CH_3_		C-11, C-12, C-13
OMe-2	3.26 s	50.7, CH_3_		C-2
OH-13	2.42 br d (5.6)		H-13	-
OH-14	2.29 br d (4.4)		H-14	-

**Table 3:** ^1^H (400 MHz, CDCl_3_) and ^13^C (100 MHz, CDCl_3_) NMR, and COSY, HMBC data for **3**.

Position	δ_H_ (*J* in Hz)	δ_C_, type	COSY	HMBC
1		159.8, C		
2		107.7, C		
3α/β	1.76 d (14.4); 3.56 d (14.4)	39.6, CH_2_		C-1, C-2, C-4, C-5, C-18
4		131.0, C		
5	5.29 d (8.4)	133.5, CH	H-6	C-3, C-18
6	4.86 ddd (12.0, 8.4, 3.2)	68.7, CH	H-5, H_2_-7	-
7/7′	1.34 m; 1.80 m	44.9, CH_2_	H-6, H-8	C-5, C-6, C-8, C-9, C-19
8	1.21 m	35.3, CH	H_2_-7, H-9, H_3_-19	-
9	3.65 ddd (10.0, 10.0, 4.4)	87.3, CH	H-8, H_2_-10	-
10/10′	1.38 m; 2.05 m	32.8, CH_2_	H-9, H_2_-11	C-9, C-11, C-12
11/11′	1.61 m; 2.22 dd (13.2, 8.0)	35.5, CH_2_	H_2_-10	C-9, C-12, C-13, C-20
12		85.1, C		
13	3.40 d (8.4)	76.7, CH	OH-13	C-1, C-12, C-20
14	4.75 d (5.2)	63.5, CH	OH-14	C-1, C-2, C-12, C-13, C-15
15		128.5, C		
16	2.08 s	9.6, CH_3_		C-1, C-15, C-17
17		171.9, C		
18	1.83 s	24.0, CH_3_		C-3, C-4, C-5
19	0.84 d (6.8)	20.1, CH_3_	H-8	C-7, C-8, C-9
20	1.28 s	21.1, CH_3_		C-11, C-12, C-13
OMe-2	3.20 s	51.1, CH_3_		C-2
OH-13	3.05 d (8.4)		H-13	C-13, C-14
OH-14	2.70 d (5.2)		H-14	C-13, C-14

**Table 4:** Effects of **1**–**4** on LPS-induced pro-inflammatory iNOS release in RAW 264.7 cells at a concentration of 10 μM. The data presented are the relative intensity normalized to the LPS- stimulated group. Compounds **2** and **4** were found to have the higher inhibition effects on LPS- induced iNOS expression in macrophages expression.

	iNOS
	Expression (% of LPS)
LPS	100.0 ± 7.0
**1**	109.0 ± 19.2
**2**	67.7 ± 2.4
**3**	79.5 ± 9.4
**4**	61.9 ± 7.3

## Supplementary Materials

The Supplementary Materials are available online at https://www.mdpi.com/1660-3397/17/4/214/s1. ESIMS, HRESIMS, IR, 1D (^1^H NMR, ^13^C NMR, and DEPT spectra), and 2D (COSY, HSQC, HMBC, and NOESY) spectra of new compounds **1**–**3** and ^1^H and ^13^C NMR spectra of **4**.
