# Supplementary material for: New Furanocembranoids from Briareum violaceum
Source: Mar Drugs. 2019 Apr 5;17(4):214. doi: 10.3390/md17040214 (PMC6520705; doi:10.3390/md17040214)
Supplement: Supplementary file 1 [file marinedrugs-17-00214-s001.pdf]

Supporting information

Briaviodiol F (**1**)

S 1-1 ESIMS spectrum of **1** 2
S 1-2 HRESIMS spectrum of **1** 2
S 1-3 IR spectrum of **1** 3
S 1-4 ^{1}H NMR spectrum of **1** (400 MHz, $CDCl_3$) 3
S 1-5 ^{13}C NMR spectrum of **1** (100 MHz, $CDCl_3$) 4
S 1-6 DEPT spectrum of **1** 4
S 1-7 HSQC spectrum of **1** 5
S 1-8 HMBC spectrum of **1** 5
S 1-9 ^{1}H–^{1}H COSY spectrum of **1** 6
S 1-10 NOESY spectrum of **1** 6

Briaviotriol A (**2**)

S 2-1 ESIMS spectrum of **2** 7
S 2-2 HRESIMS spectrum of **2** 7
S 2-3 IR spectrum of **2** 8
S 2-4 ^{1}H NMR spectrum of **2** (400 MHz, $CDCl_3$) 8
S 2-5 ^{13}C NMR spectrum of **2** (100 MHz, $CDCl_3$) 9
S 2-6 DEPT spectrum of **2** 9
S 2-7 HSQC spectrum of **2** 10
S 2-8 HMBC spectrum of **2** 10
S 2-9 ^{1}H–^{1}H COSY spectrum of **2** 11
S 2-10 NOESY spectrum of **2** 11

Briaviotriol B (**3**)

S 3-1 ESIMS spectrum of **3** 12
S 3-2 HRESIMS spectrum of **3** 12
S 3-3 IR spectrum of **3** 13
S 3-4 ^{1}H NMR spectrum of **3** (400 MHz, $CDCl_3$) 13
S 3-5 ^{13}C NMR spectrum of **3** (100 MHz, $CDCl_3$) 14
S 3-6 DEPT spectrum of **3** 14
S 3-7 HSQC spectrum of **3** 15
S 3-8 HMBC spectrum of **3** 15
S 3-9 ^{1}H–^{1}H COSY spectrum of **3** 16
S 3-10 NOESY spectrum of **3** 16

Briaviodiol A (**4**)

S 4-1 ^{1}H NMR spectrum of **4** (400 MHz, $CDCl_3$) 17
S 4-2 ^{13}C NMR spectrum of **4** (100 MHz, $CDCl_3$) 17

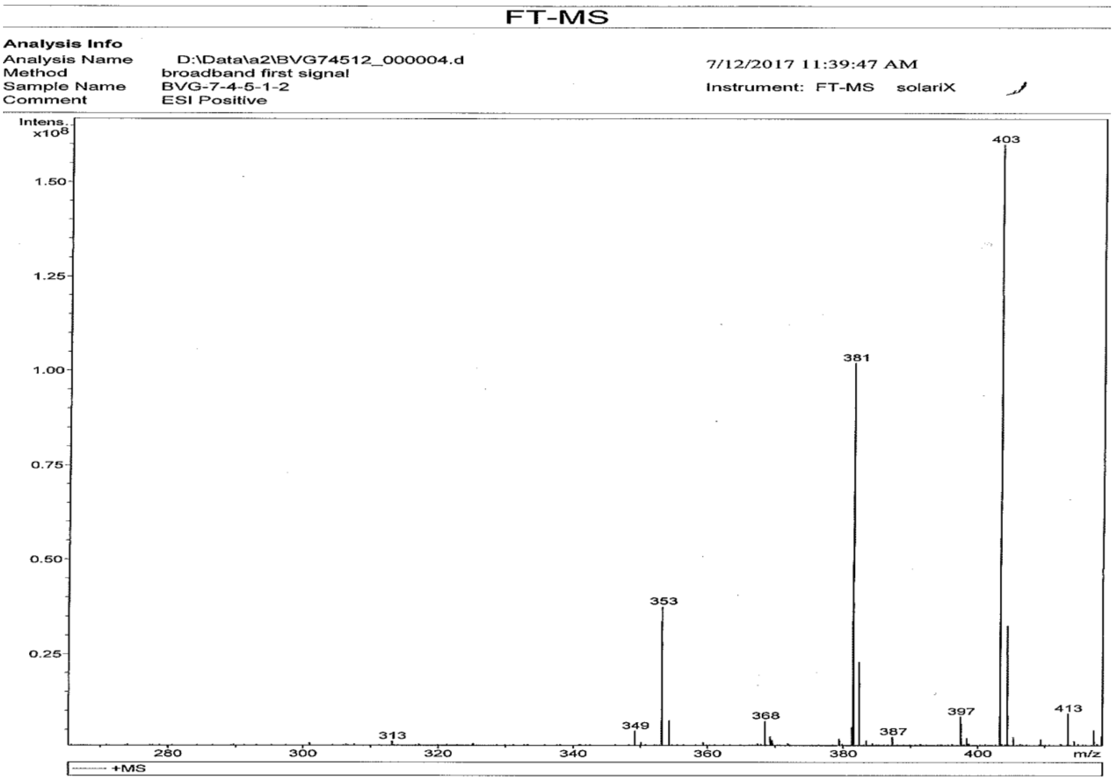

S 1-1 ESIMS spectrum of **1**

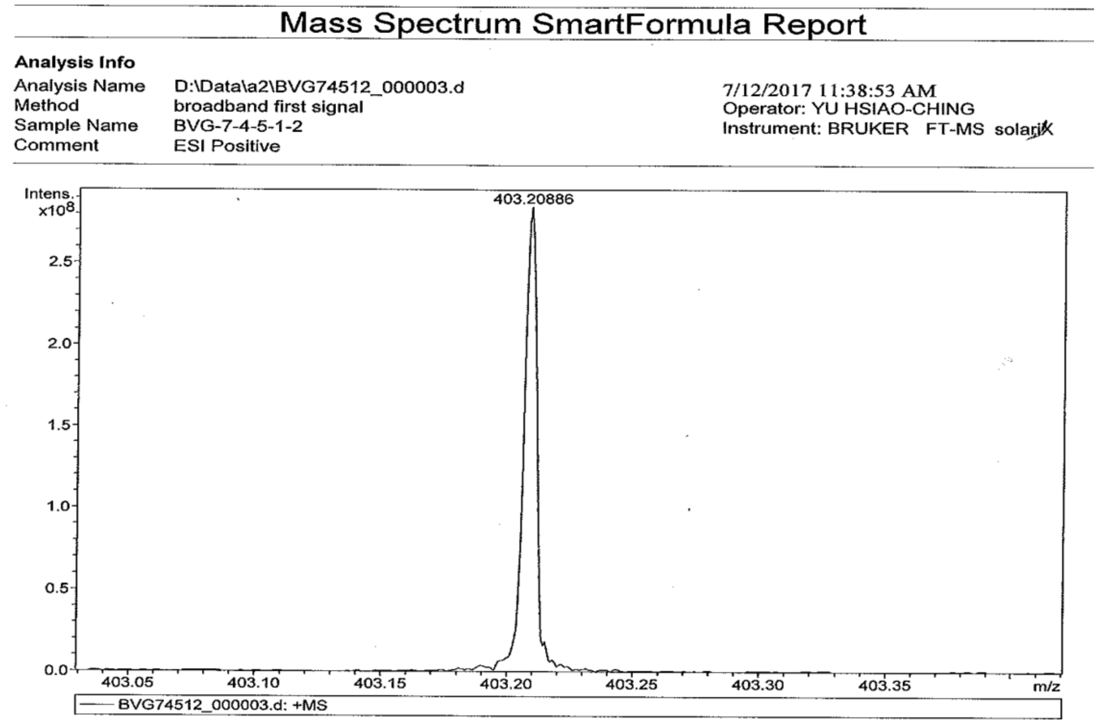

Meas. m/z	#	Formula	Score	m/z	err [mDa]	err [ppm]	mSigma	rdb	e⁻ Conf	N-Rule
403.20886	1	C 21 H 32 Na O 6	100.00	403.20911	0.25	0.62	11.5	5.5	even	ok

S 1-2 HRESIMS spectrum of **1**

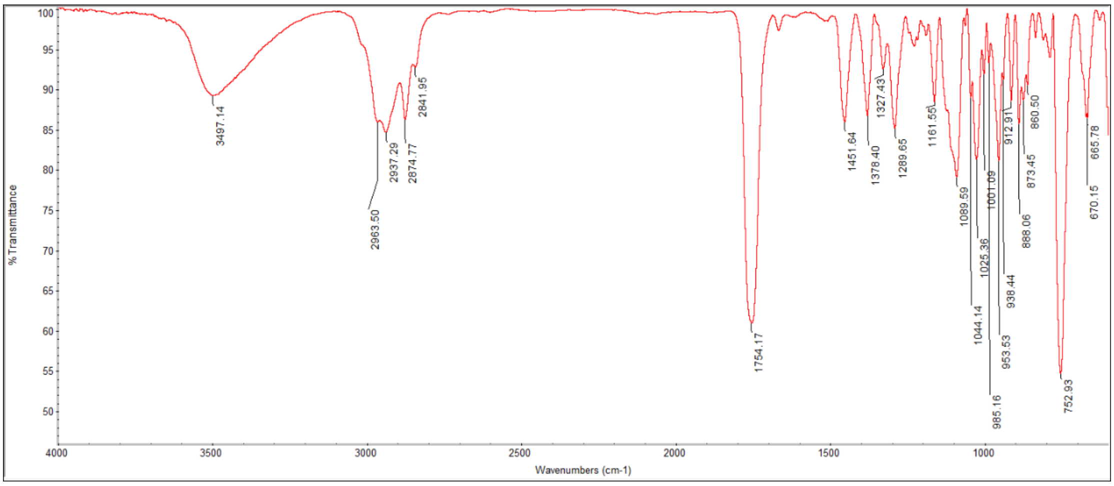

S 1-3 IR spectrum of **1**

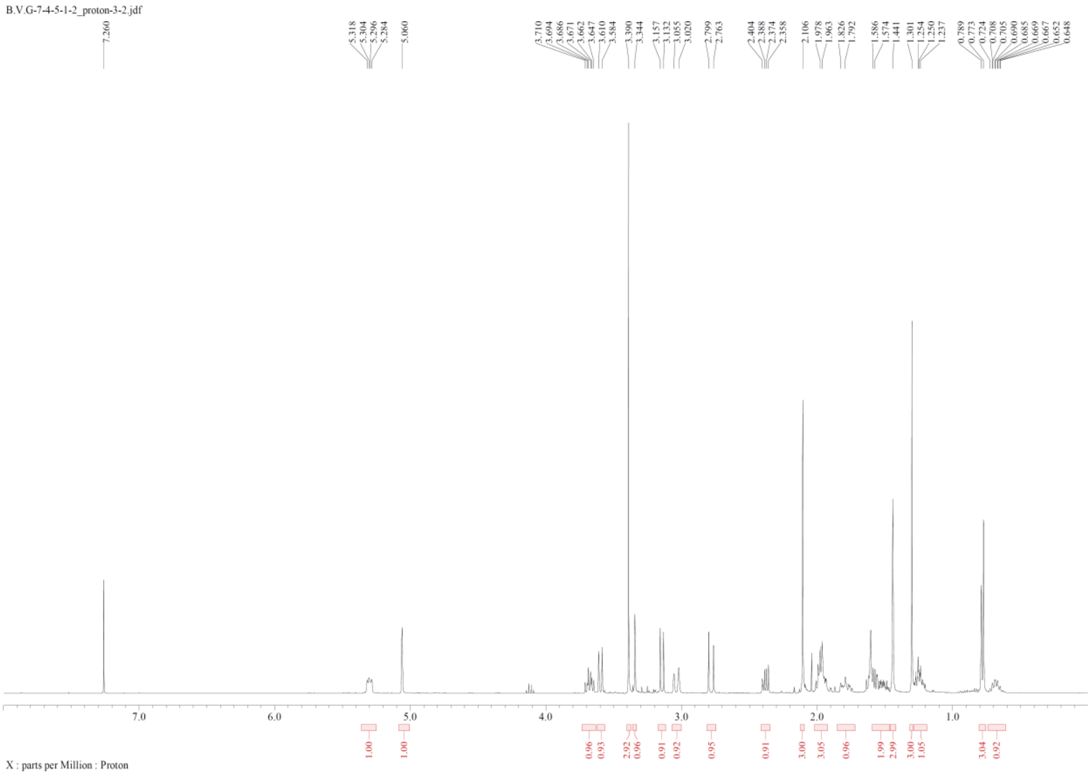

S 1-4 ^{1}H NMR spectrum of **1** (400 MHz, $CDCl_3$)

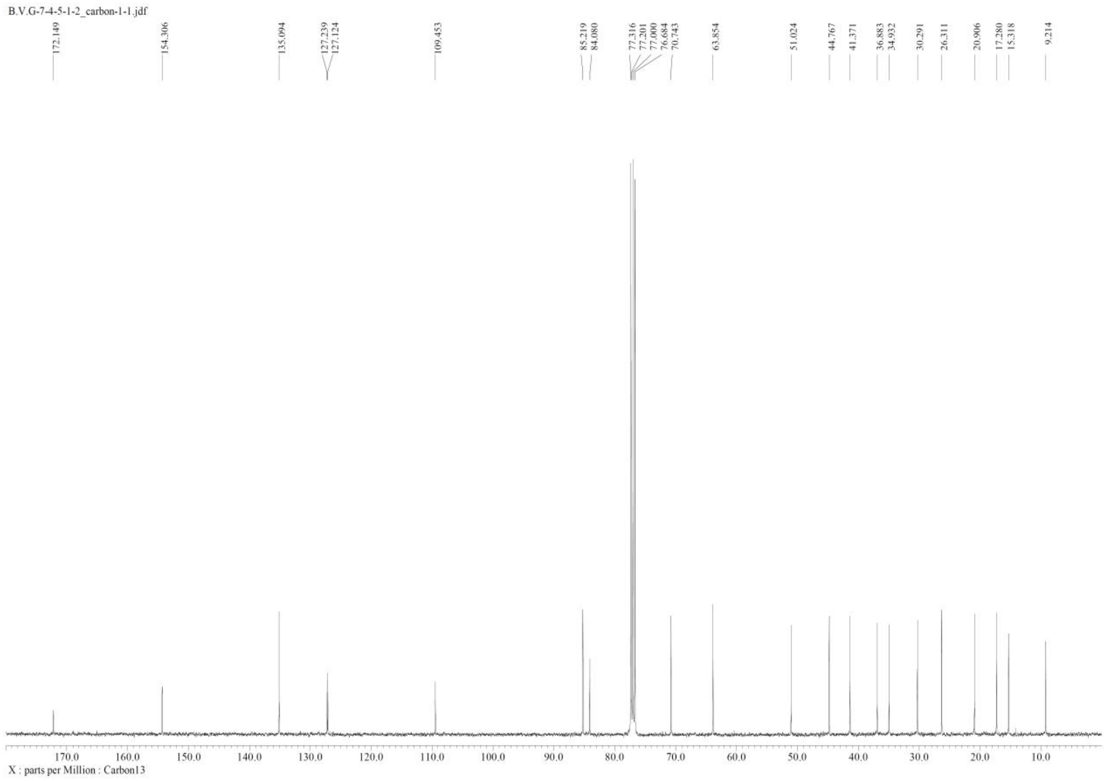

S 1-5 ^{13}C NMR spectrum of **1** (100 MHz, $CDCl_3$)

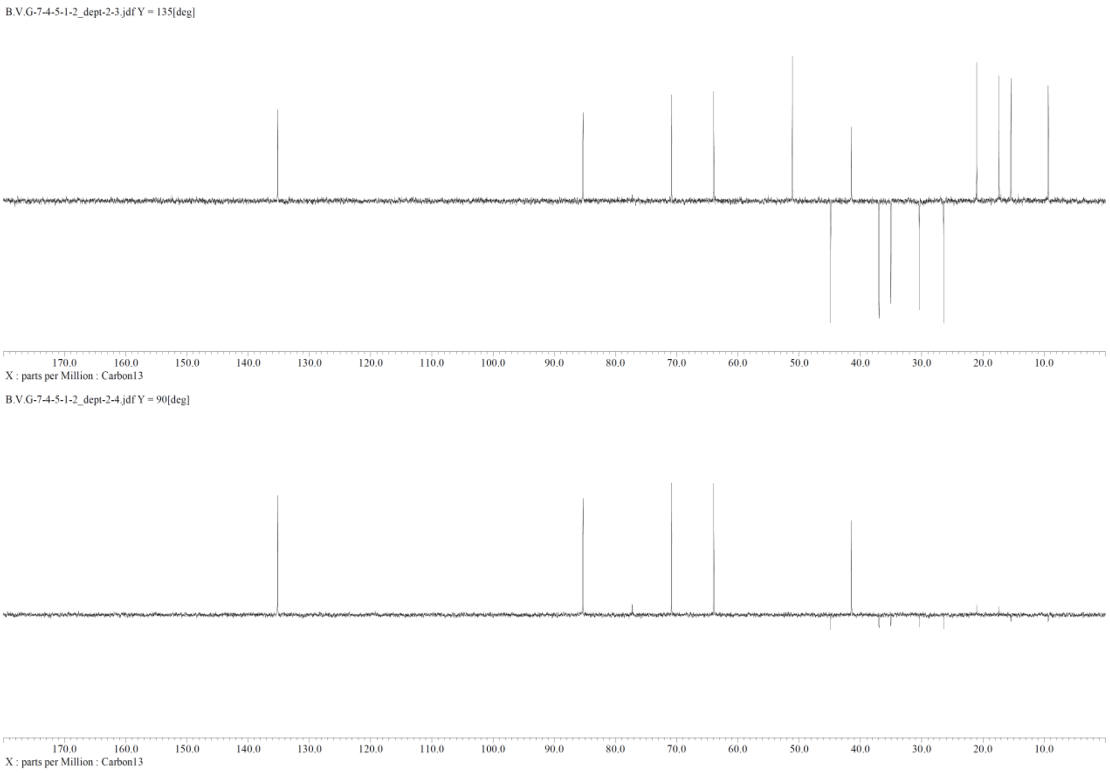

S 1-6 DEPT spectrum of **1**

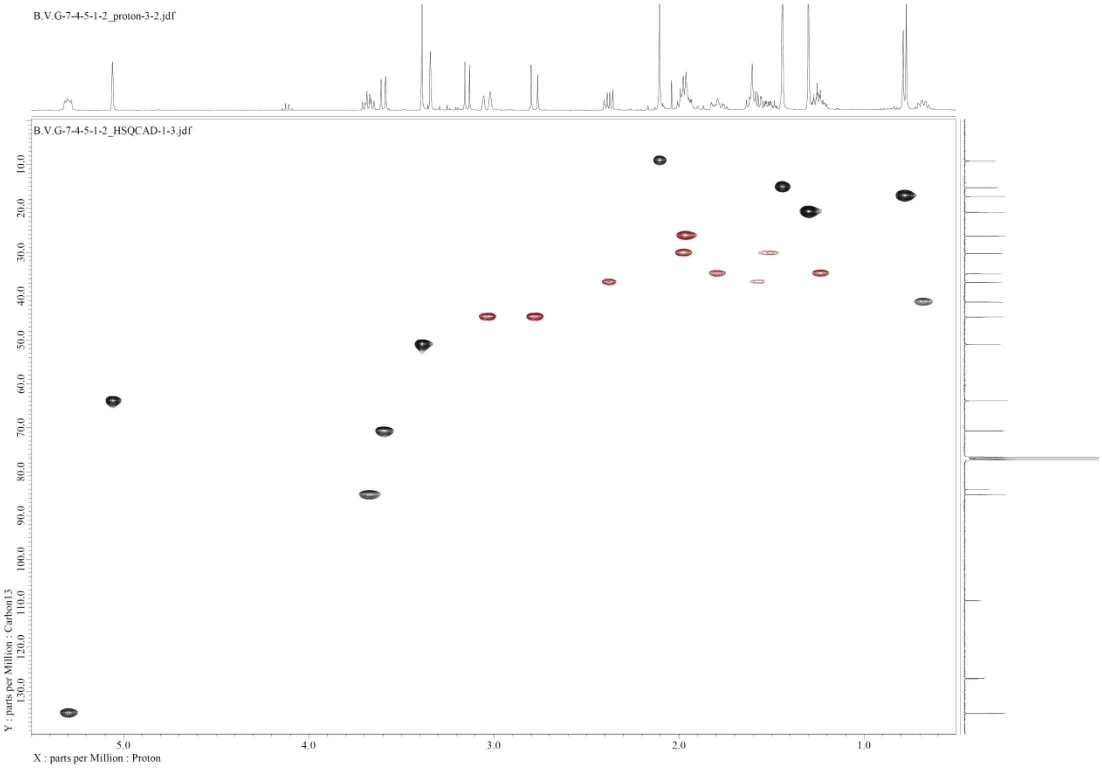

S 1-7 HSQC spectrum of **1**

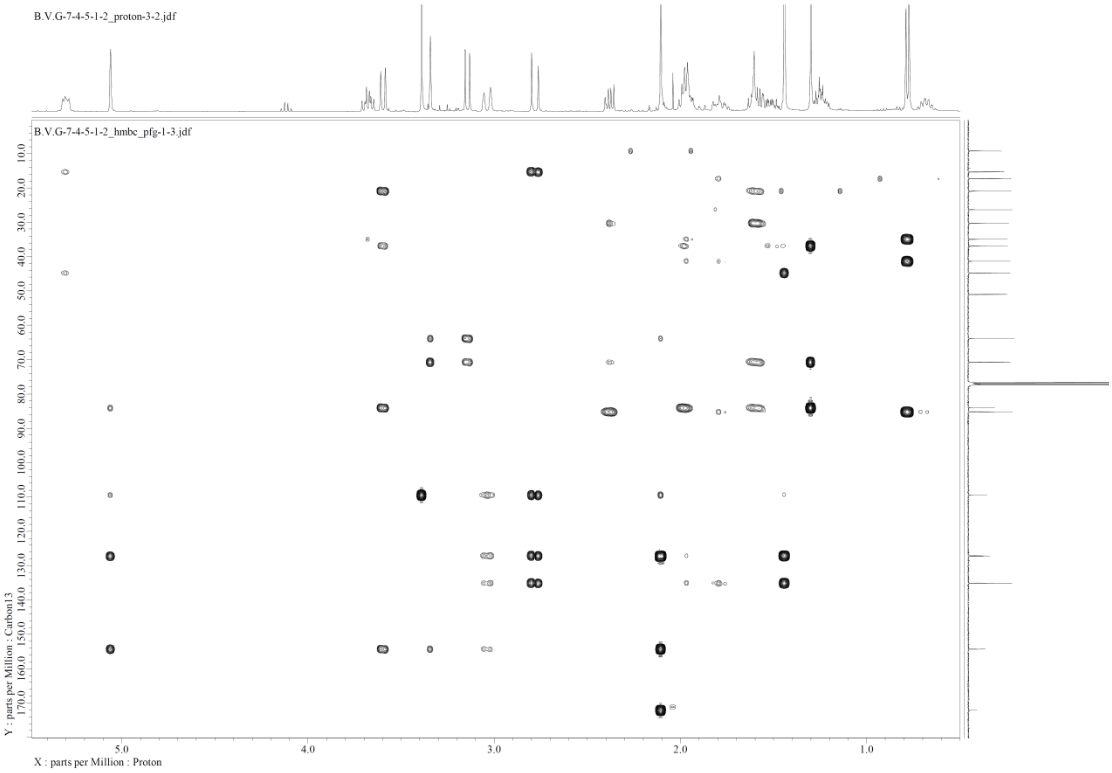

S 1-8 HMBC spectrum of **1**

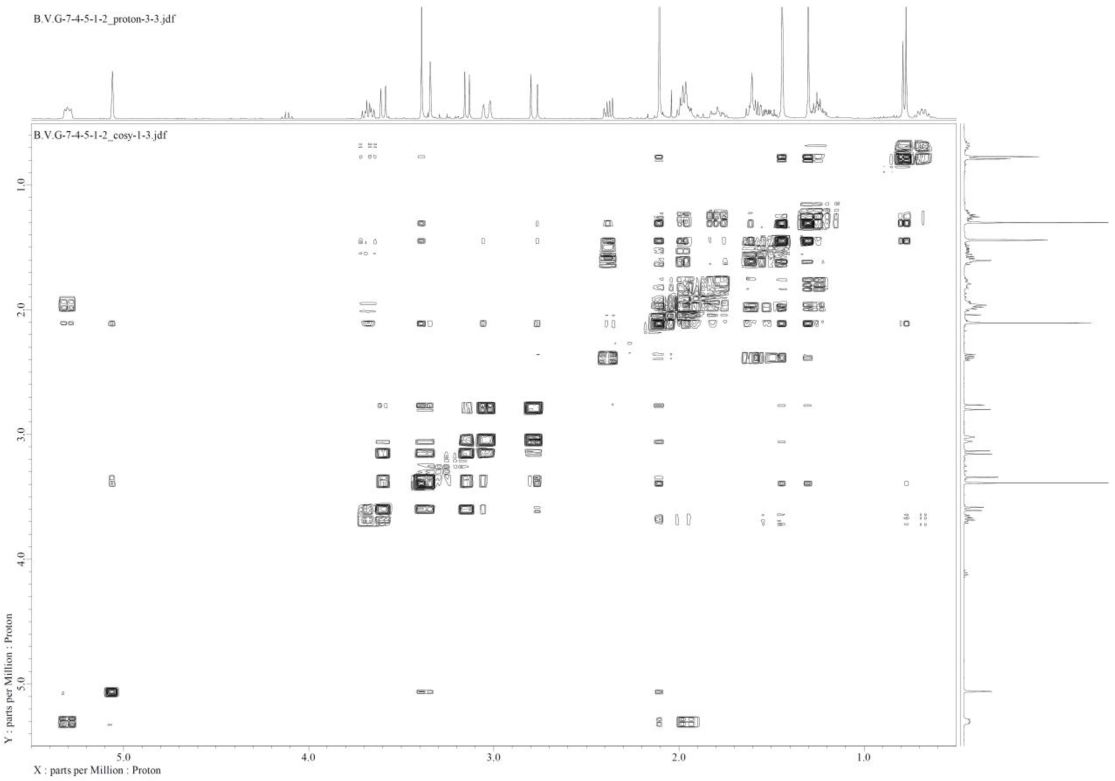

S 1-9 ^{1}H–^{1}H COSY spectrum of **1**

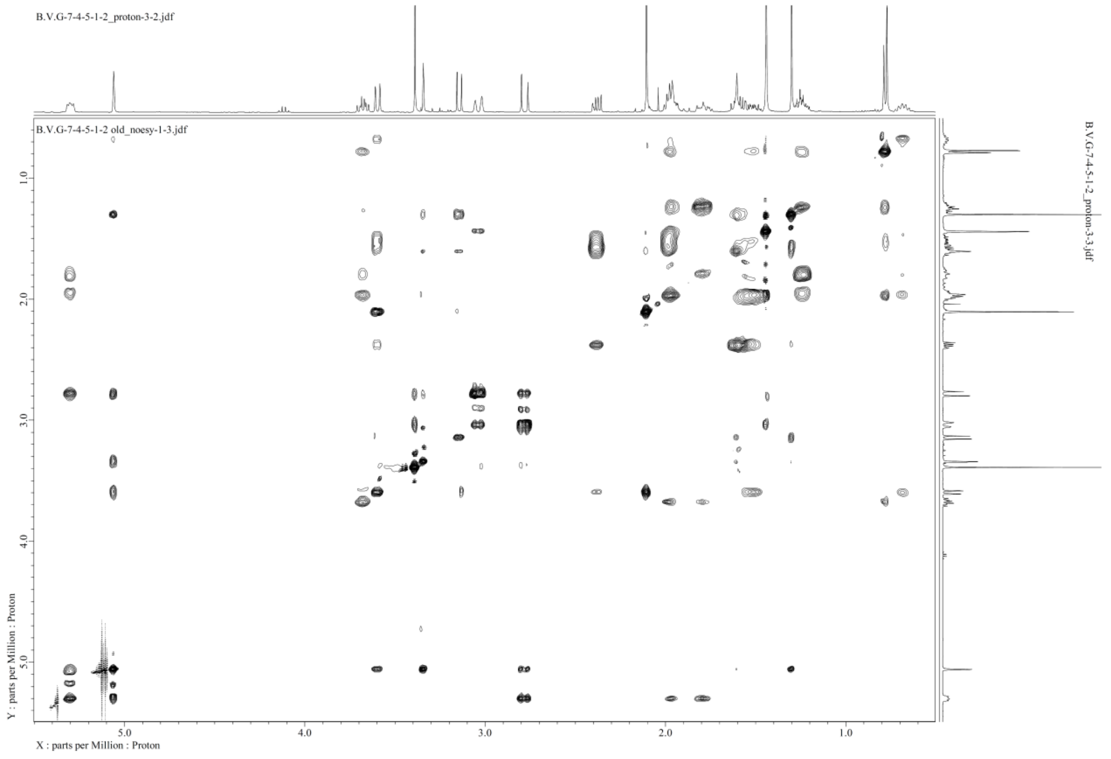

S 1-10 NOESY spectrum of **1**

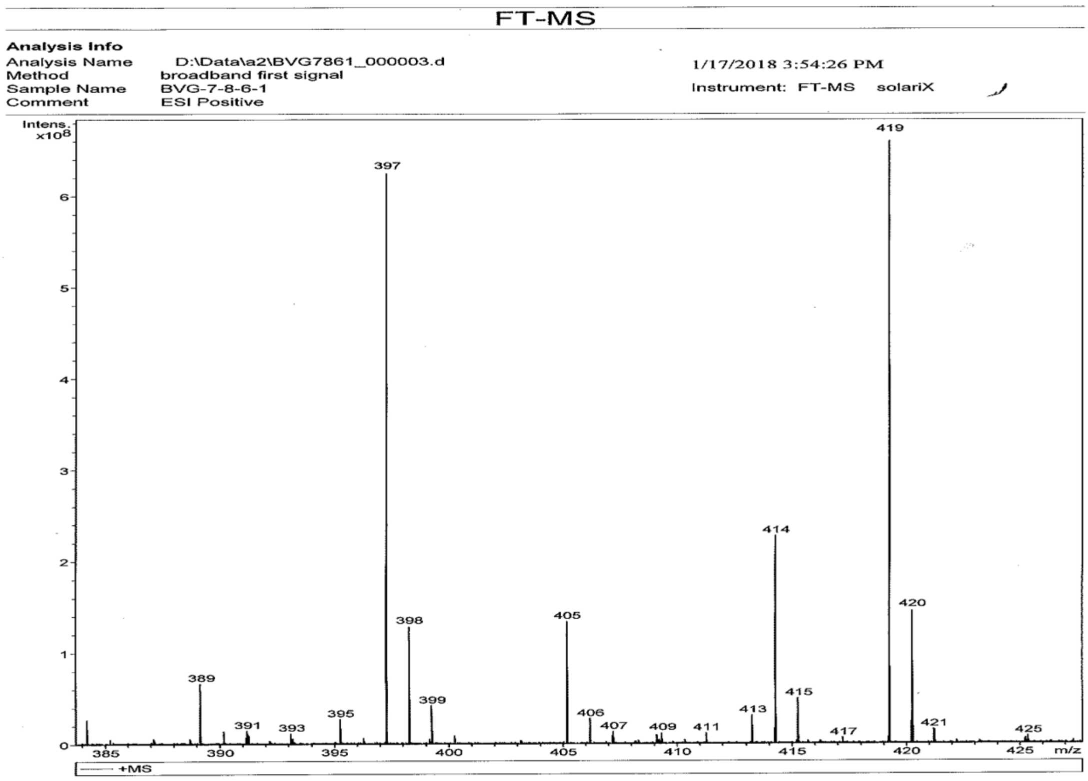

S 2-1 ESIMS spectrum of **2**

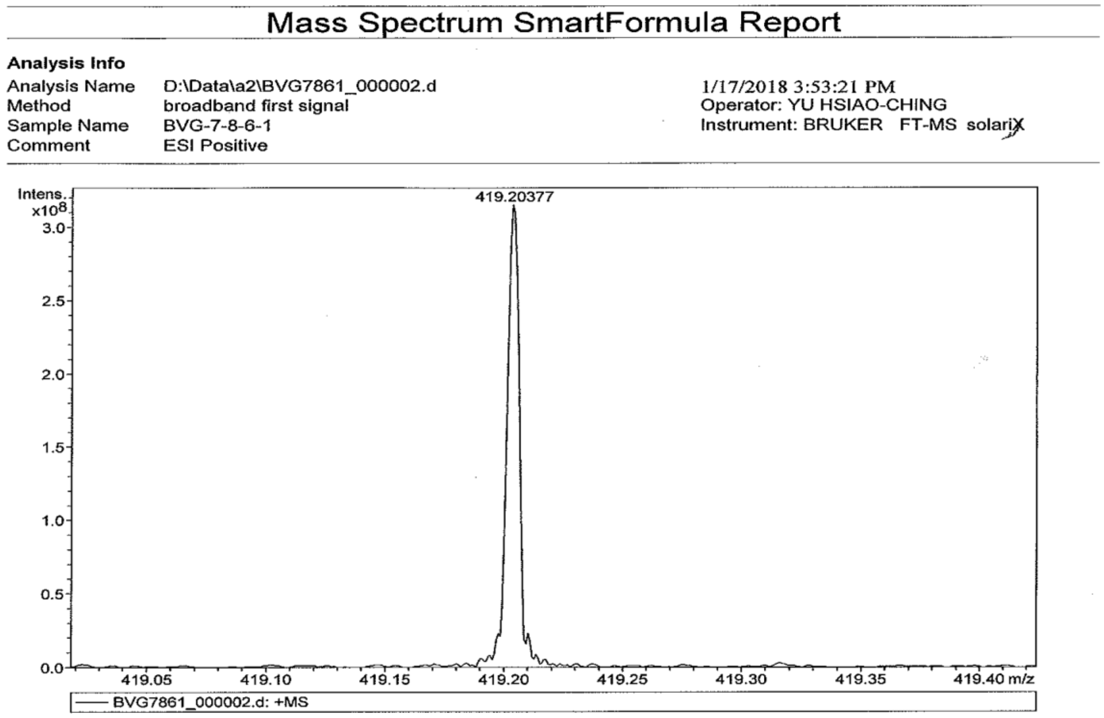

Meas. m/z	#	Formula	Score	m/z	err [mDa]	err [ppm]	mSigma	rdb	e⁻ Conf	N-Rule
419.20377	1	C 21 H 32 Na O 7	100.00	419.20402	0.25	0.60	14.8	5.5	even	ok

S 2-2 HRESIMS spectrum of **2**

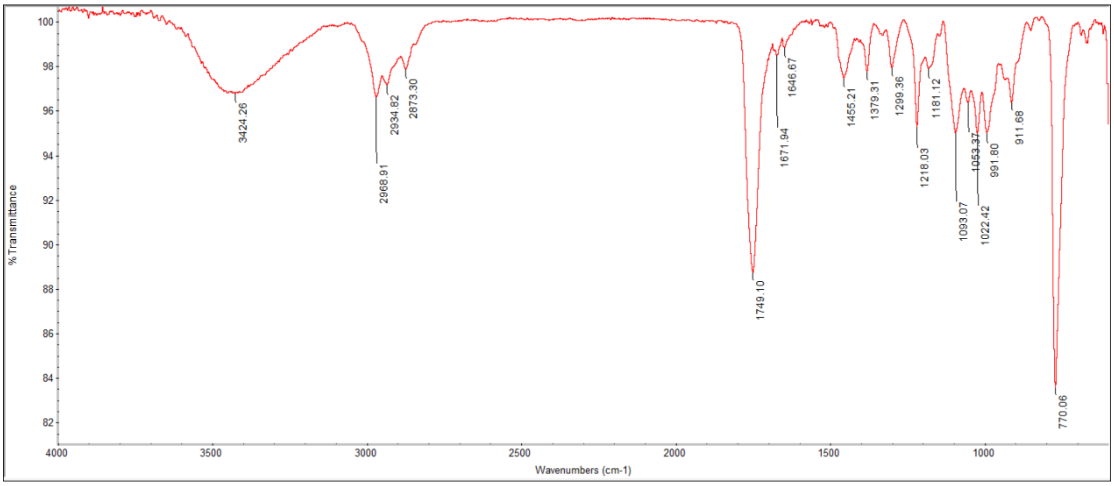

S 2-3 IR spectrum of **2**

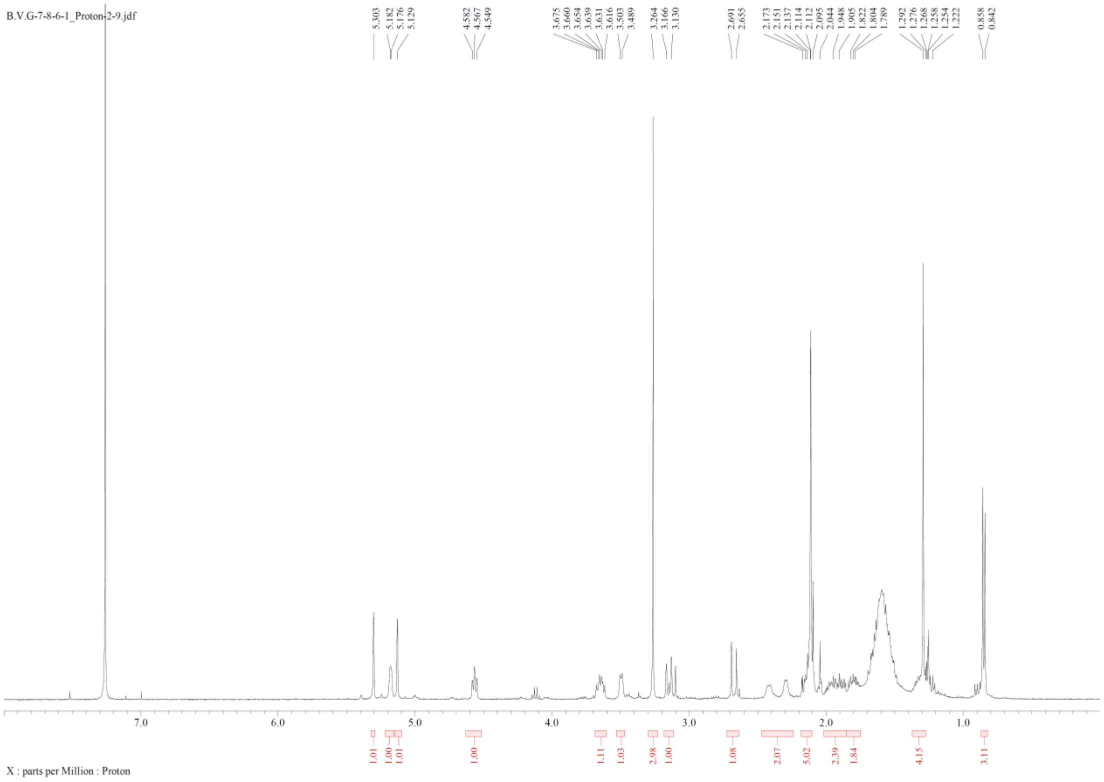

S 2-4 ^{1}H NMR spectrum of **2** (400 MHz, $CDCl_3$)

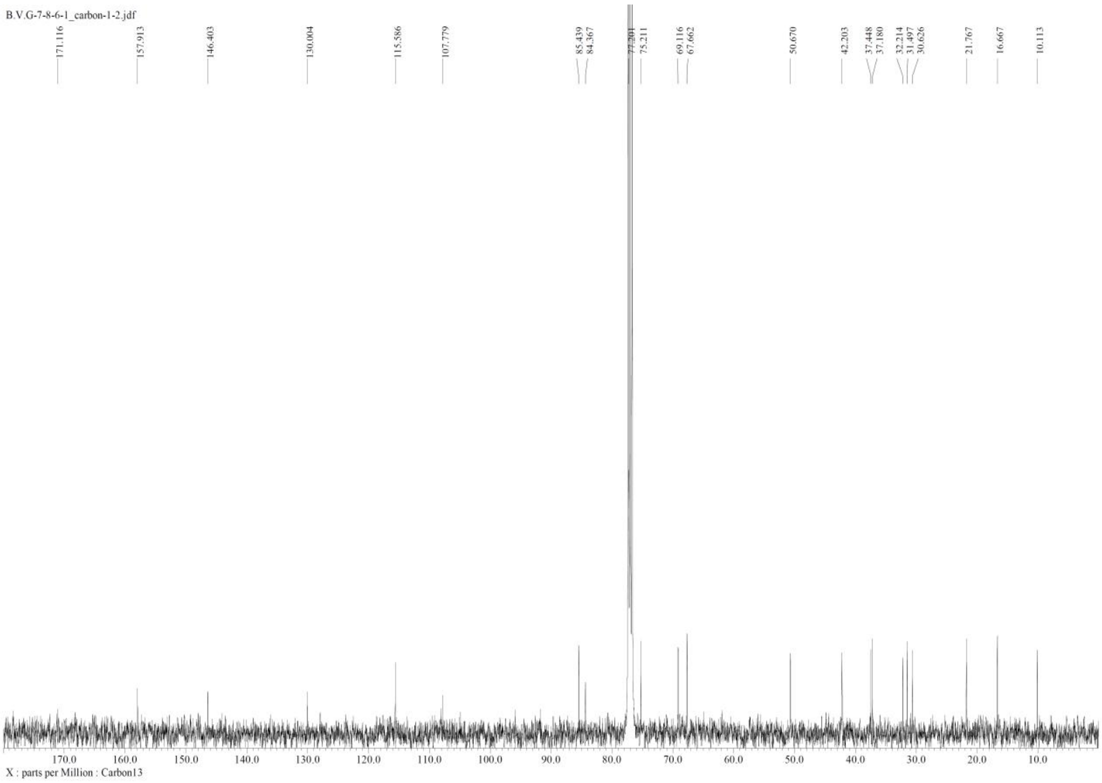

S 2-5 ^{13}C NMR spectrum of **2** (100 MHz, $CDCl_3$)

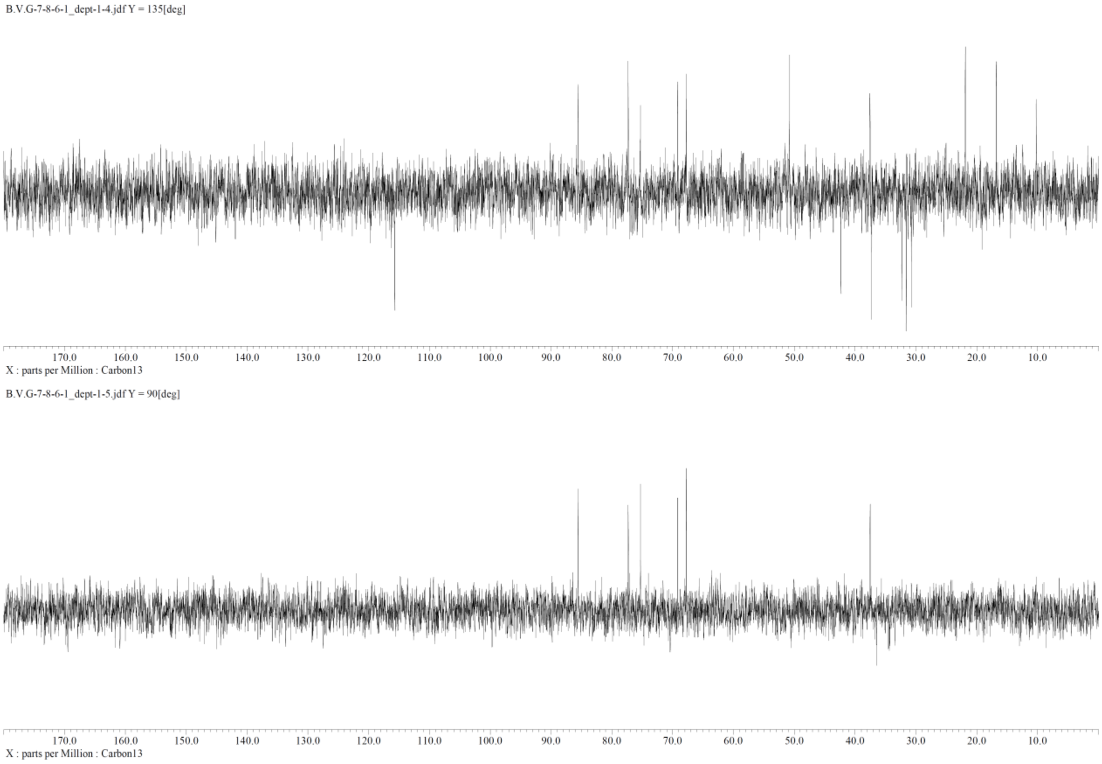

S 2-6 DEPT spectrum of **2**

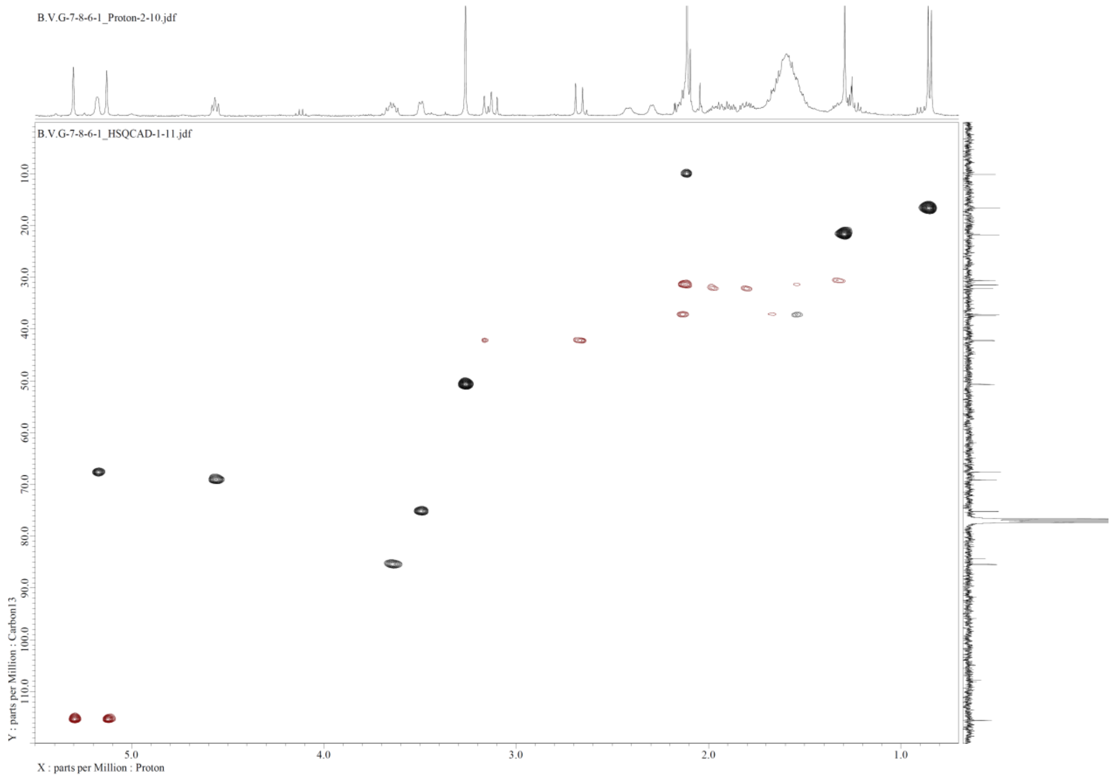

S 2-7 HSQC spectrum of **2**

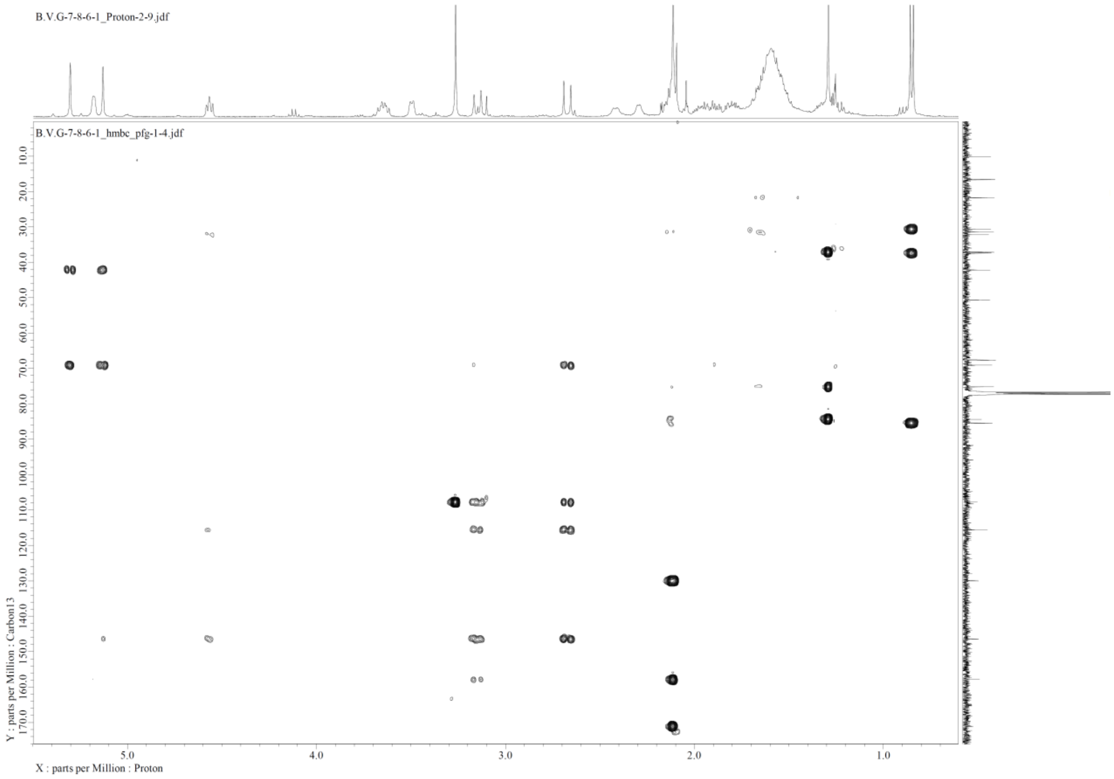

S 2-8 HMBC spectrum of **2**

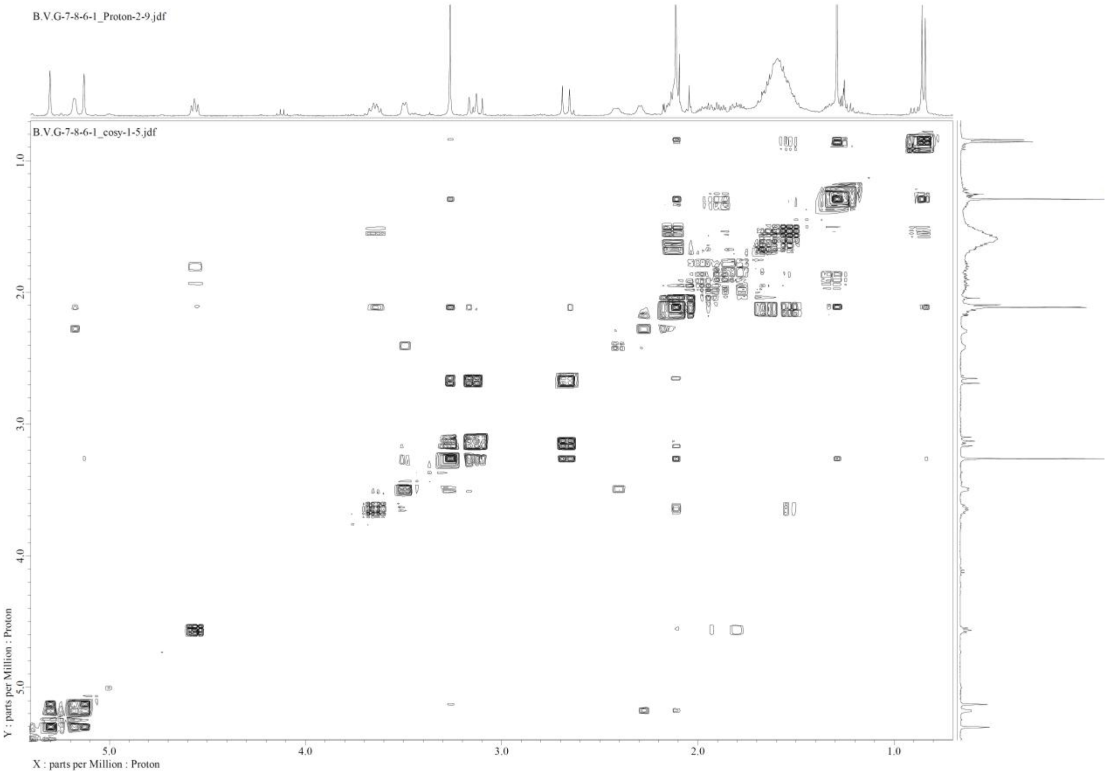

S 2-9 ^{1}H–^{1}H COSY spectrum of **2**

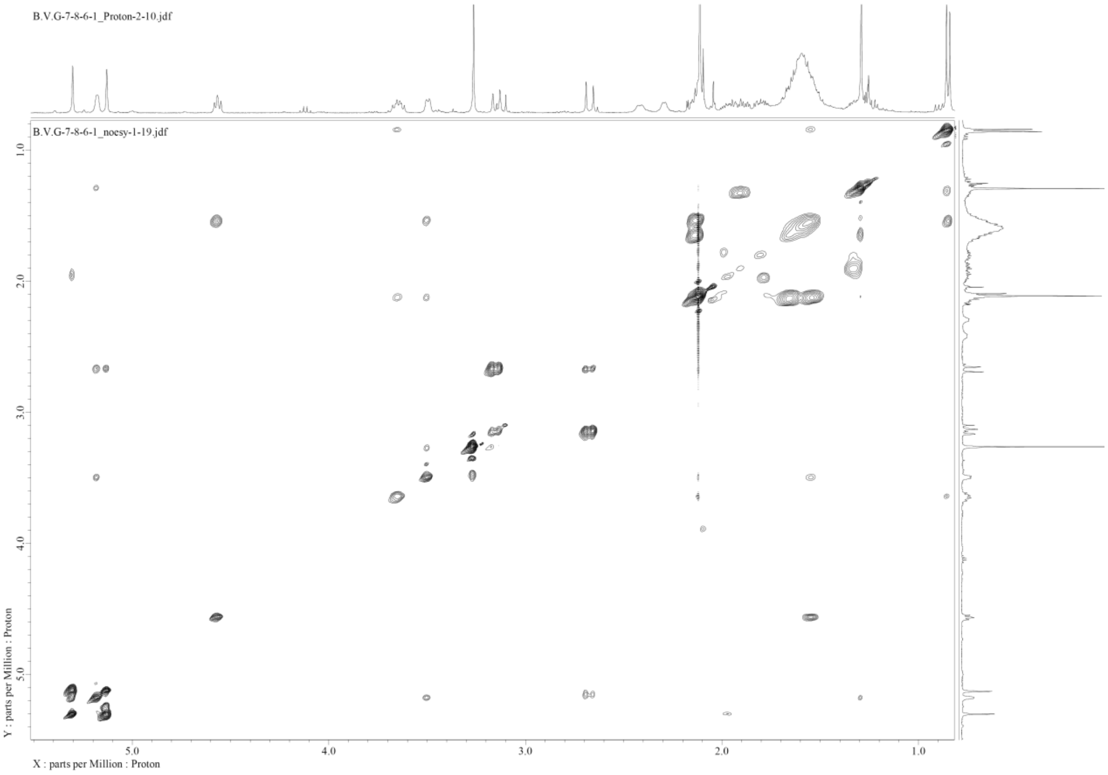

S 2-10 NOESY spectrum of **2**

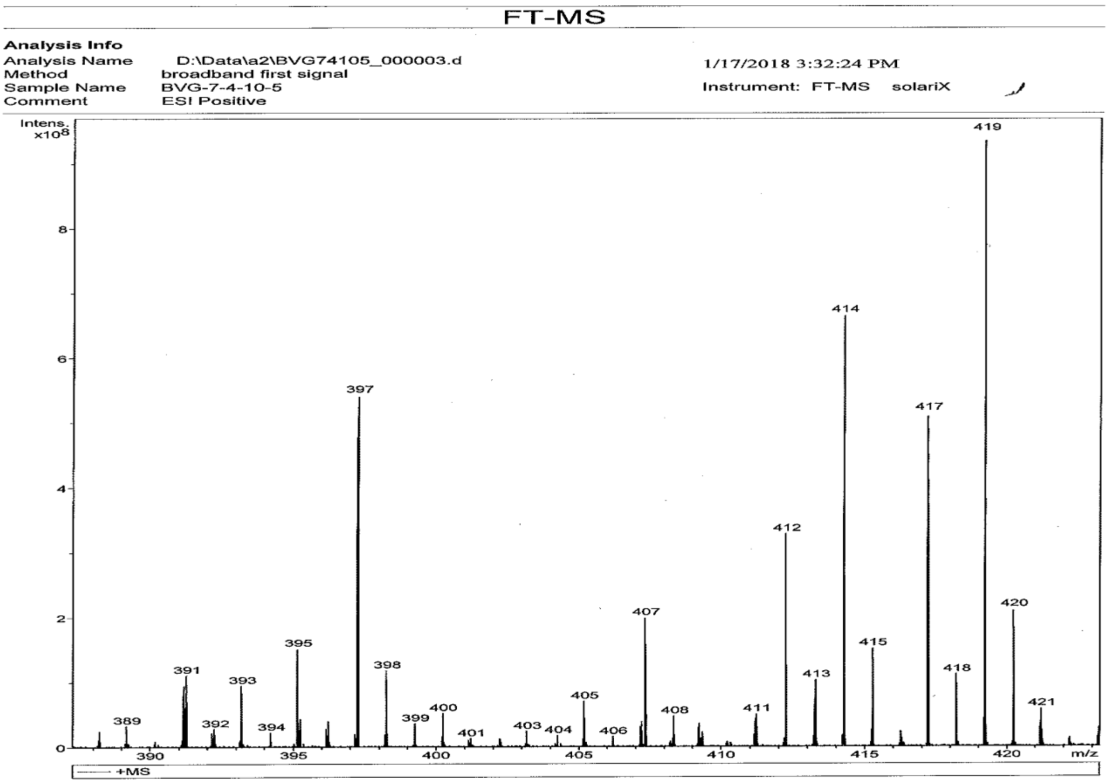

S 3-1 ESIMS spectrum of **3**

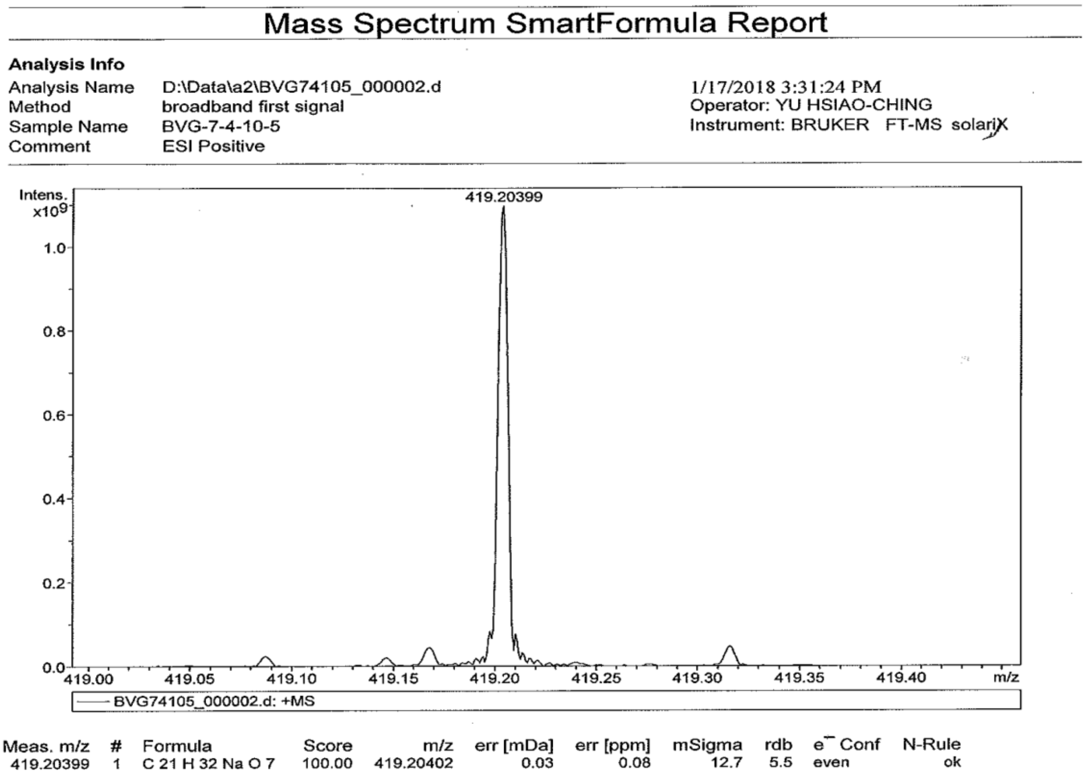

S 3-2 HRESIMS spectrum of **3**

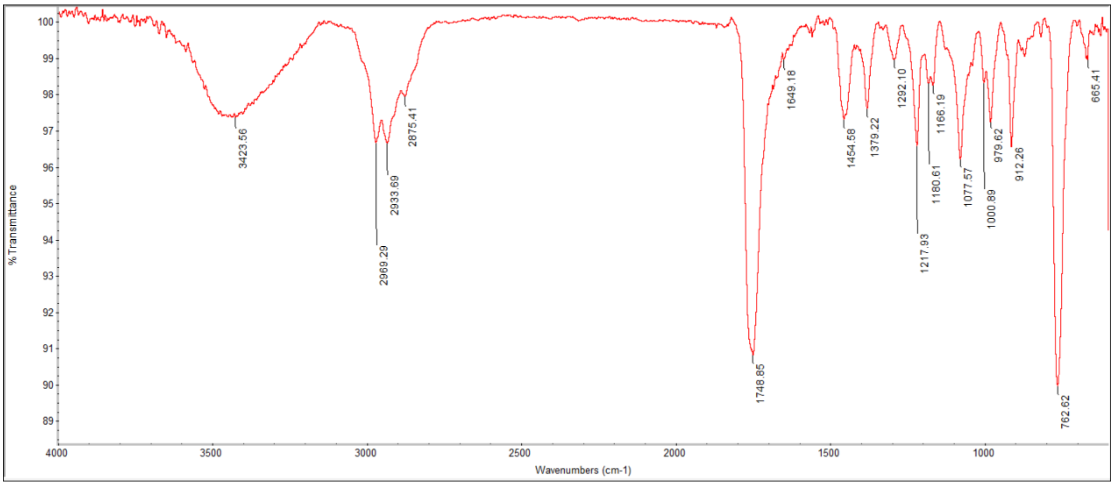

S 3-3 IR spectrum of **3**

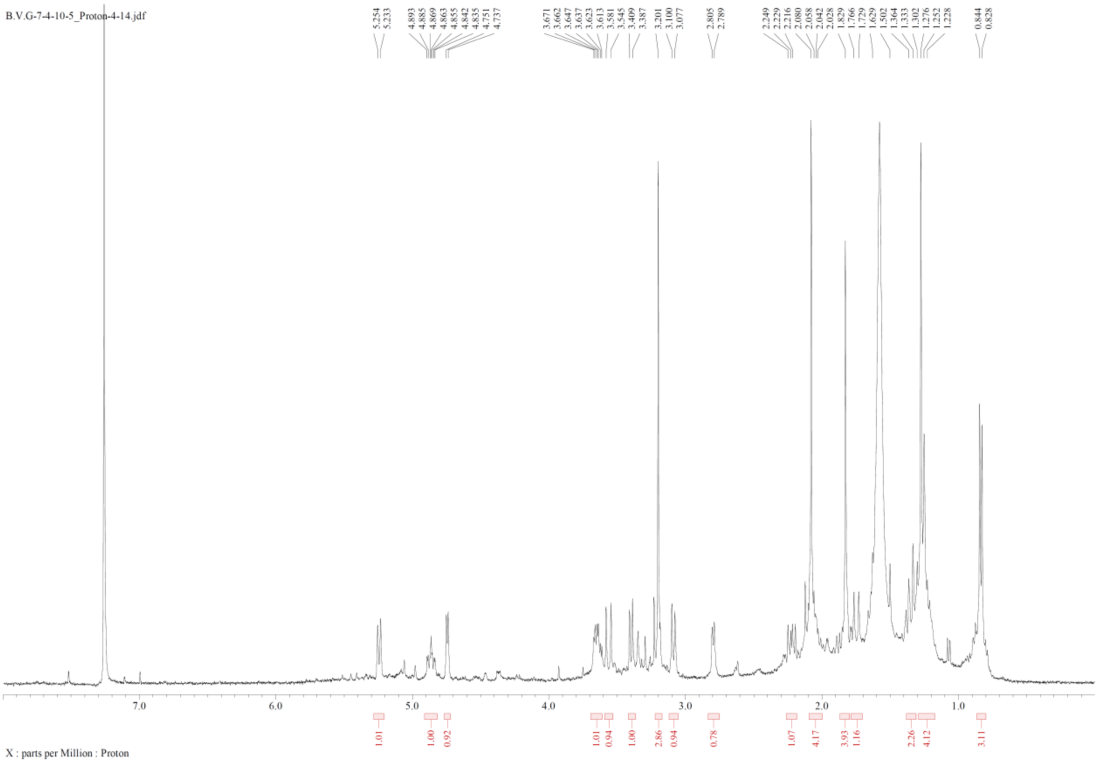

S 3-4 ^{1}H NMR spectrum of **3** (400 MHz, $CDCl_3$)

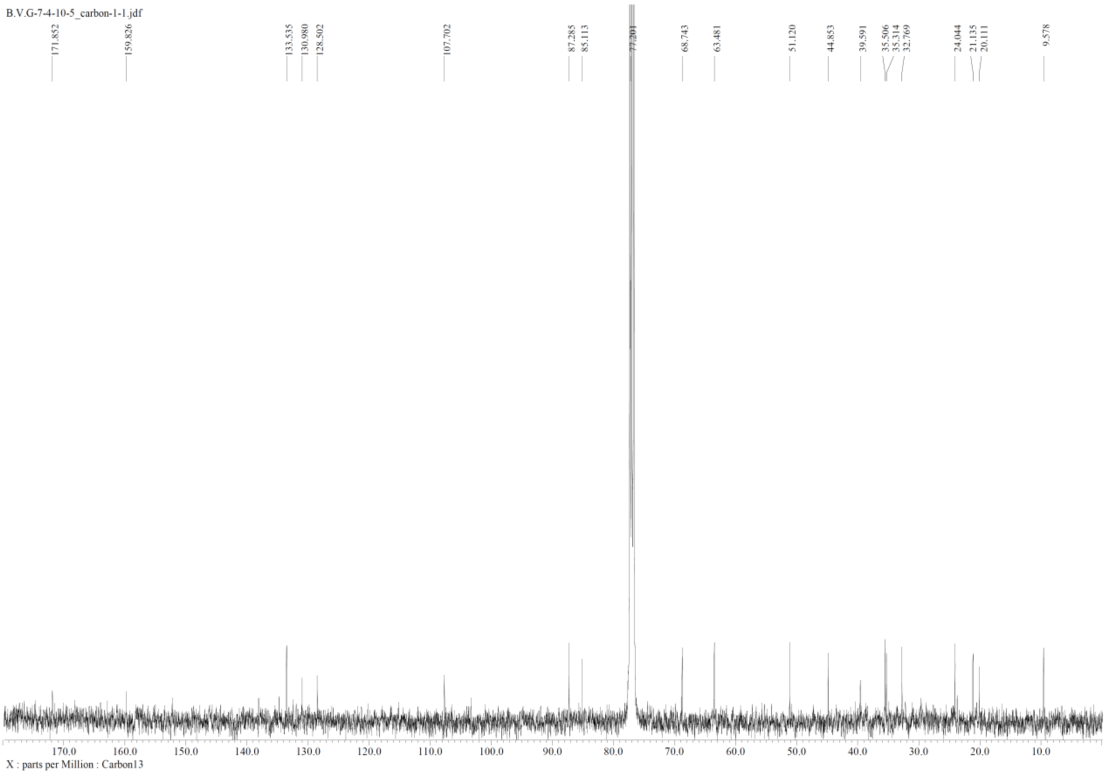

S 3-5 ^{13}C NMR spectrum of **3** (100 MHz, $CDCl_3$)

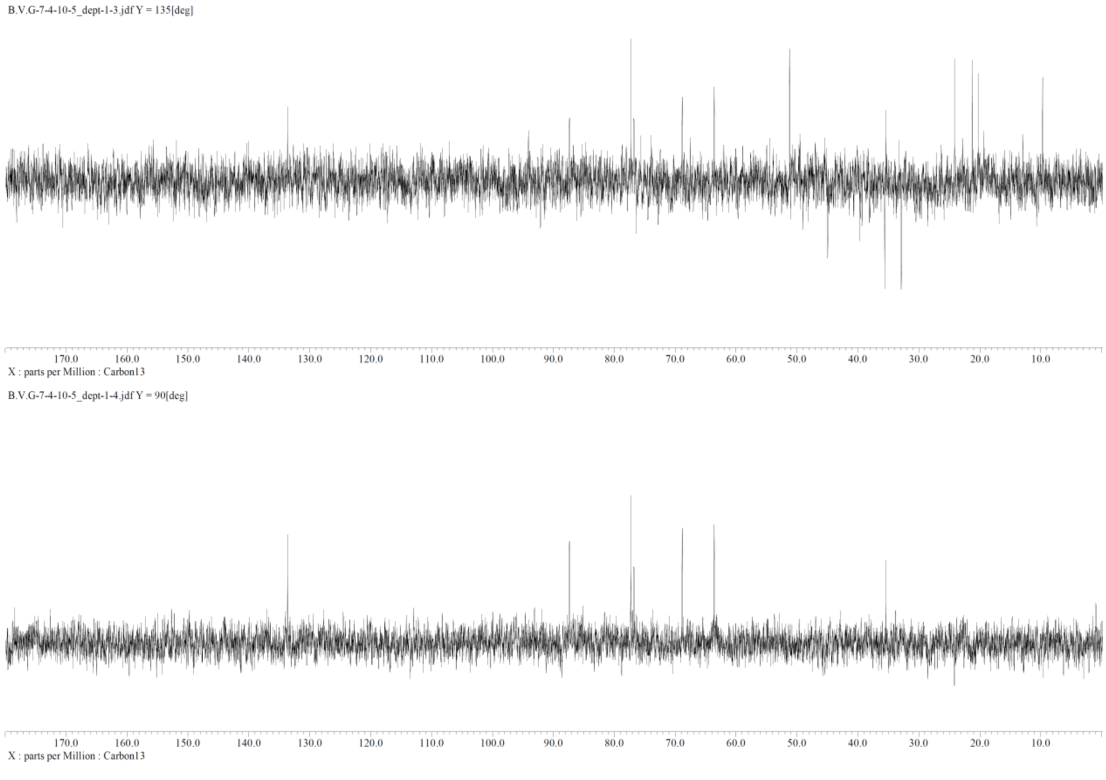

S 3-6 DEPT spectrum of **3**

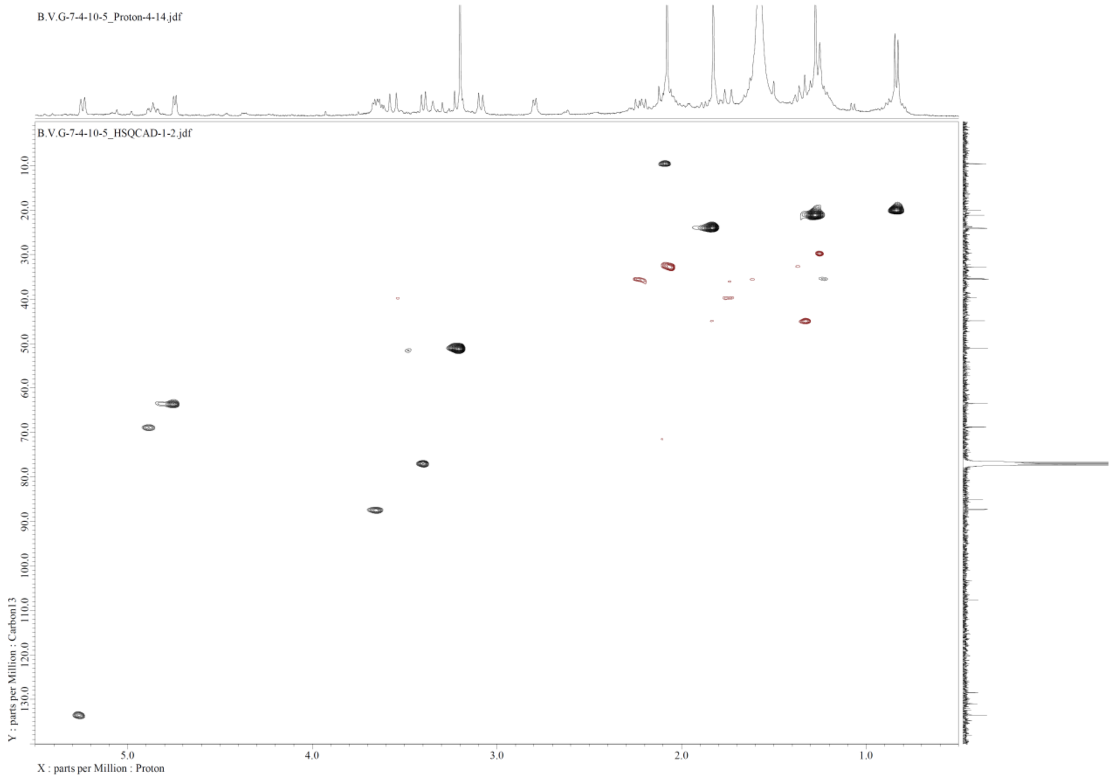

S 3-7 HSQC spectrum of **3**

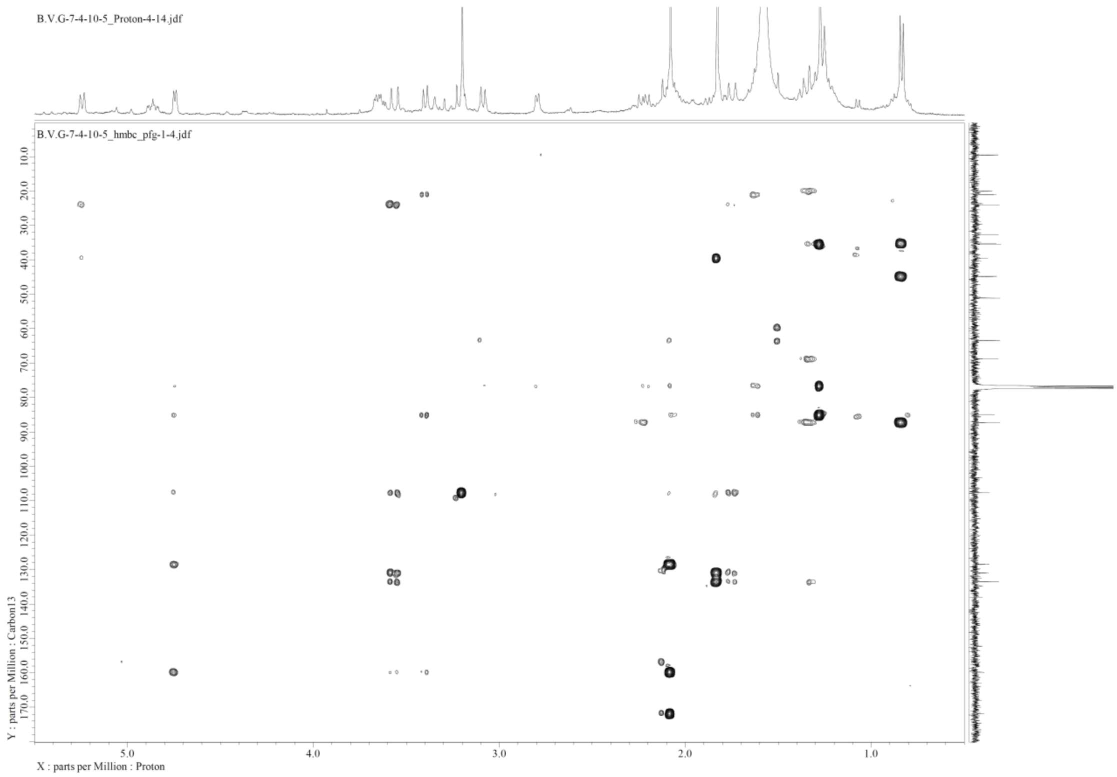

S 3-8 HMBC spectrum of **3**

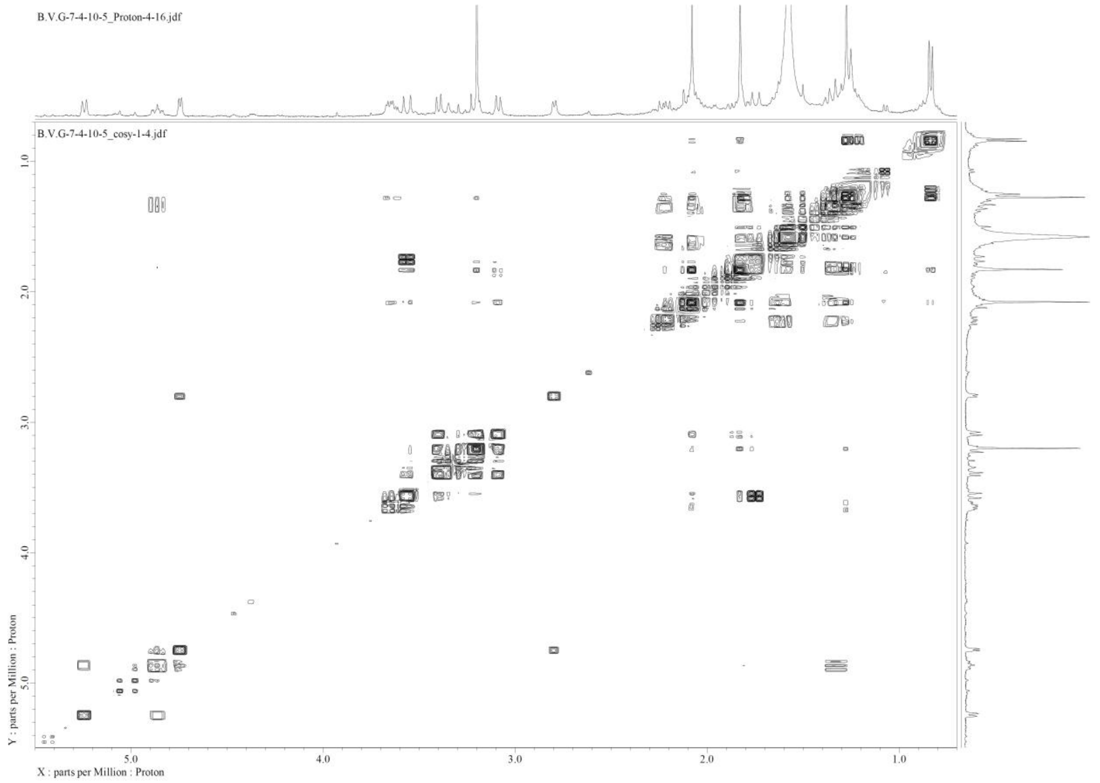

S 3-9 ^{1}H–^{1}H COSY spectrum of **3**

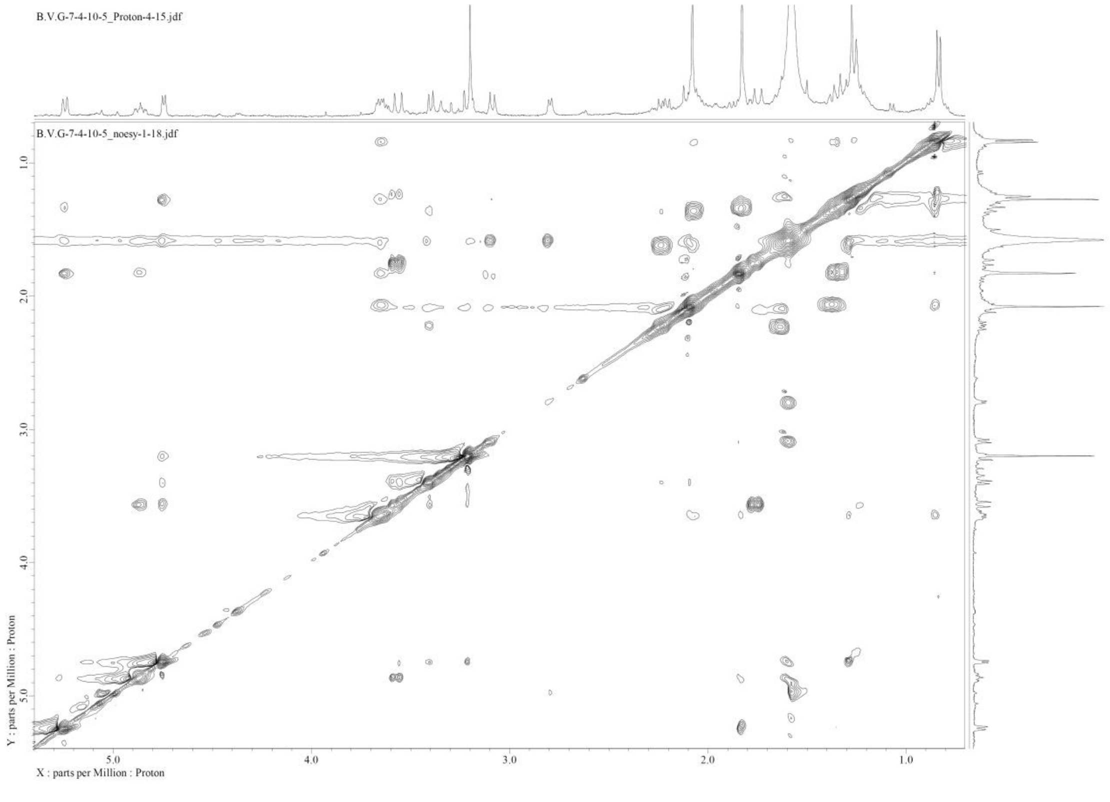

S 3-10 NOESY spectrum of **3**

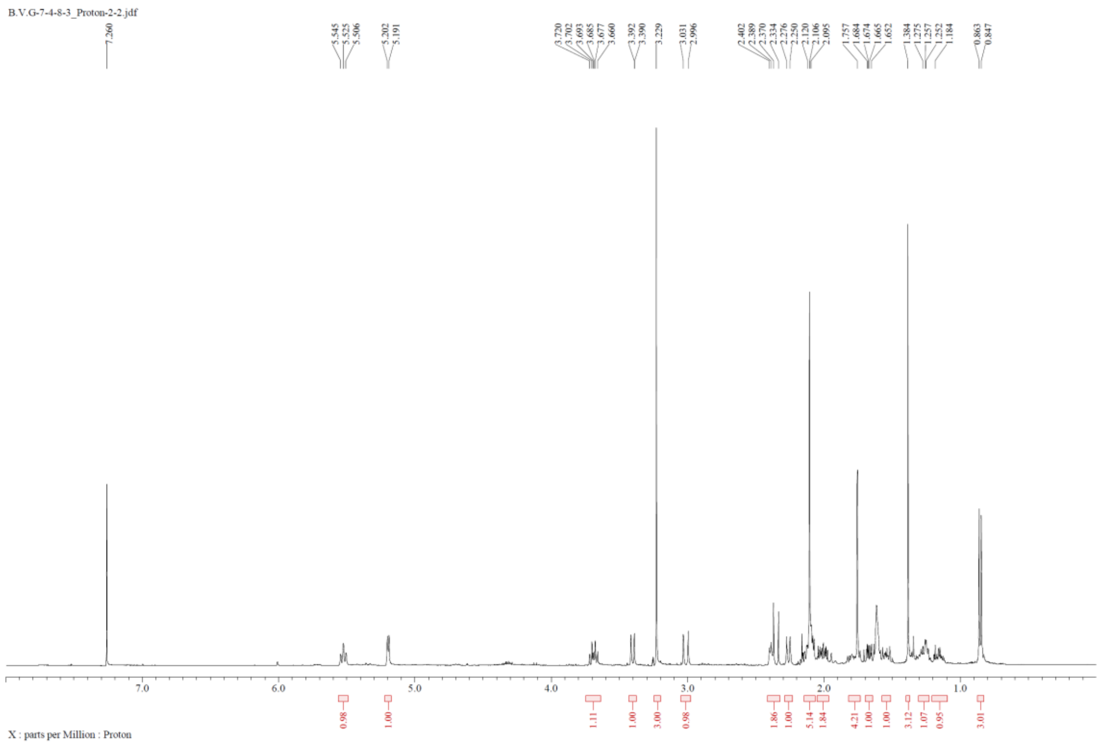

S 4-1 ^{1}H NMR spectrum of **4** (400 MHz, $CDCl_3$)

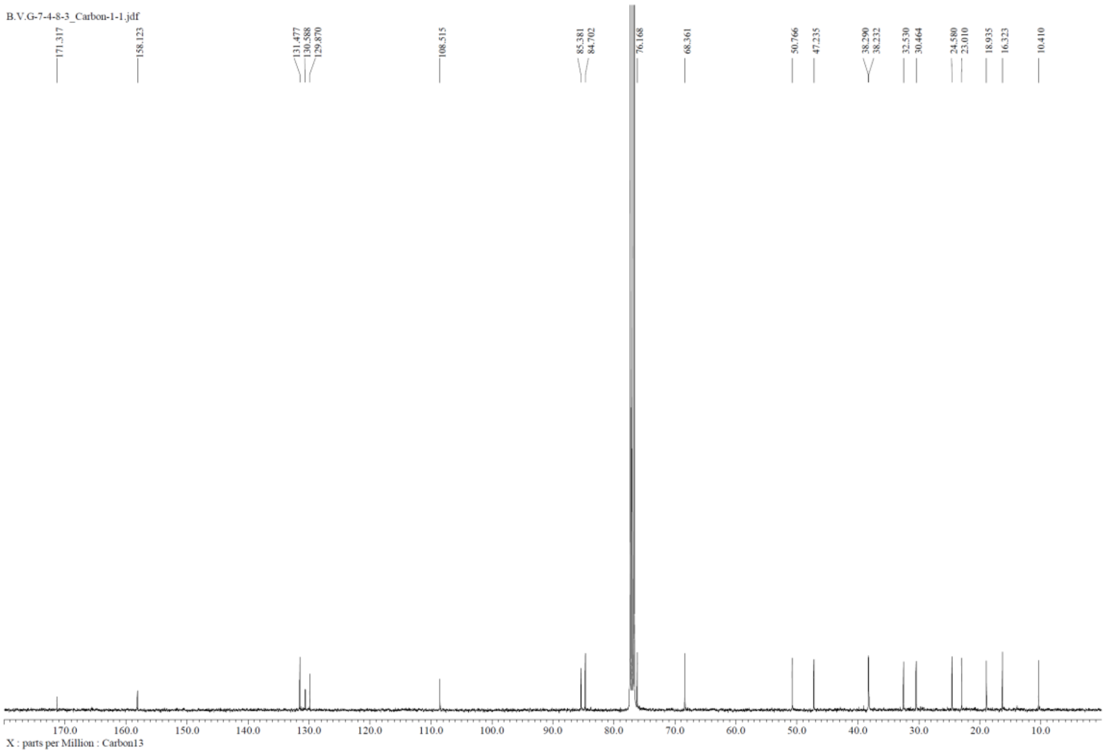

S 4-2 ^{13}C NMR spectrum of **4** (100 MHz, $CDCl_3$)
